# Mistreatment in Residency: Intervening With the REWIND Communication Tool

**DOI:** 10.15766/mep_2374-8265.11245

**Published:** 2022-04-26

**Authors:** Joyce Pang, Natasha Navejar, John Paul Sánchez

**Affiliations:** 1 Third-Year Resident, Department of Surgery, University of New Mexico School of Medicine; 2 Fourth-Year Medical Student, Baylor College of Medicine; 3 Professor and Vice Chair, Department of Emergency Medicine, University of New Mexico School of Medicine; Executive Associate Vice Chancellor, Health Sciences Center Office for Diversity, Equity, and Inclusion, University of New Mexico School of Medicine

**Keywords:** Mistreatment, Case-Based Learning, Diversity, Inclusion, Health Equity, Anti-racism

## Abstract

**Introduction:**

There is a lack of curricula addressing the alarming rates of resident physician mistreatment. As the ACGME works to address diversity, equity, and inclusion in GME, there has been increasing attention paid to the issue of mistreatment. Previous studies have noted a high prevalence of mistreatment within GME. Despite this, there are few published interventions to address the mistreatment of residents. We developed a workshop for residents to provide an overview of mistreatment in residency and teach them REWIND (relax, express, why, inquire, negotiate, determine), a communication tool to address mistreatment directly.

**Methods:**

We designed a 60-minute workshop for residents with didactics on mistreatment in GME, followed by three case discussions. Four case scenarios were developed to represent different types of mistreatment and situations. We implemented the workshop twice and asked participants to self-rate proficiency around the workshop objectives with pre- and postsurveys.

**Results:**

A total of 11 GME learners completed both the pre- and postsurveys between the two workshop implementations. GME learners who responded demonstrated significantly higher self-rated proficiency on each objective postworkshop compared to preworkshop (*p* < .05). Free responses on the survey demonstrated that participants particularly enjoyed the case discussions and wanted more practice with REWIND.

**Discussion:**

Our workshop improved participant self-rated proficiency around the mistreatment of resident physicians. The workshop can be used in the future as part of a multifaceted institutional response to mistreatment.

## Educational Objectives

By the end of this activity, learners will be able to:

1. Define the most common forms of resident physician mistreatment.
2. Demonstrate the use of the REWIND (relax, express, why, inquire, negotiate, determine) communication tool to address mistreatment.
3. Adapt strategies to address experienced or observed mistreatment.

## Introduction

Despite literature on mistreatment in the medical learning environment having been present since the 1980s, there continues to be a lack of curricula addressing the alarming rates of resident physician mistreatment. The ACGME has focused increasing attention on the issue of mistreatment as it works to address diversity, equity, and inclusion (DEI) in its programs.^1,2^ As GME programs are asked to align with UME on DEI and mistreatment in learning environments, UME policies and interventions have served as paradigms for developing similar comprehensive plans for addressing mistreatment in GME.

Studies within GME have found that prevalence of mistreatment is high. Hu and colleagues noted that the proportion of residents reporting any mistreatment included nearly 50% of 7,409 surgery residents surveyed.^3^ At an institutional level, another recent study noted that 65% of general surgery residents surveyed had experienced mistreatment.^4^ The consequences of these rates of mistreatment are severe: Residents who have been personally mistreated are “eight times more likely to report burnout” and “four times more likely to report symptoms of anxiety and depression.”^5^ The highest sources of mistreatment vary between studies but have included patients/patients’ families, staff, faculty, other residents, and students.^3,4^ Mistreatment has also been found to be associated with burnout and suicidal thoughts.^3^ This is extremely concerning, especially since residents are already at higher risk for depression compared to the general population and suicide remains a leading cause of death for residents.^6–8^

Despite identification of the high numbers of residents who have experienced mistreatment, there are few best practices for addressing mistreatment within GME programs. When considering interventions for mistreatment in GME, the multiple roles that residents play must be accounted for. Not only do residents have patient care responsibilities, they also serve as teachers to medical students while being learners themselves.^9^ Therefore, mistreatment in GME deserves a tailored approach when developing interventions.

Within *MedEdPORTAL*, a search for resources addressing mistreatment, harassment, or microaggressions involving learners and trainees revealed a total of 13 publications.^10–22^ Of these, nine work to address medical student mistreatment.^10–12,14,16,17,19,21,22^ Of the four remaining, three are directed towards a general audience of trainees (medical students and resident physicians) and faculty, and the fourth is directed towards faculty, highlighting the use of the ERASE approach (expect that mistreatment will occur, recognize episodes of mistreatment, address the situation in real time, support the learner after the event, establish/encourage a positive culture) to address patient mistreatment of trainees.^13,15,18,20^ Of these four modules addressing a facet of resident mistreatment, three are workshops, and one is an activity utilizing forum theater. All use cases or scenarios to demonstrate the concepts they are teaching and have educational objectives focused on being able to describe and apply the knowledge that participants gain. Of the four modules, three address mistreatment of learners and faculty by patients, and only one, that of Sotto-Santiago and colleagues,^15^ addresses general mistreatment, in the form of racism, discrimination, and microaggressions in the learning environment. Sotto-Santiago and colleagues’ workshop aims for participants to “describe concepts associated with RDM [racism, discrimination, and microaggressions]” and to “practice the OWTFD (observe/why?/think/feel/desire) communication tool as a response tool to RDM,” combining both didactic learning and application within the workshop.^15^

We aimed to produce a workshop for residents that would provide a definition of mistreatment, facts about mistreatment, and a communication tool residents could use to directly address mistreatment. Existing workshops in *MedEdPORTAL* are not specifically focused on the mistreatment of residents and seek to address specific facets of mistreatment (e.g., race-based, sexual harassment) without offering a general overview. Unlike students, who are primarily learners in the clinical environment, residents must navigate increasing patient care and teaching responsibilities while simultaneously being learners.^9^ The multiplicity of this role creates unique pressures on navigating mistreatment in the GME learning environment and is best addressed through cases and discussion germane to the GME experience. To develop our GME-focused mistreatment workshop, we utilized Kern's six-step approach to curriculum development to create our educational activity.^23^

For the first step as outlined by Kern, problem identification and needs assessment, we conducted a literature review in PubMed, Google Scholar, and *MedEdPORTAL* regarding the mistreatment of resident physicians and strategies to address it. Through our literature review, we noted a high prevalence of mistreatment of resident physicians within medical education but few tools with which to address it. For step 2, in a targeted needs assessment, we identified a specific need for a workshop addressing the mistreatment of residents for residents. Such a module, beyond empowering residents, would also help institutions fulfill several major ACGME program requirements, including I.C (“recruitment and retention of a diverse and inclusive workforce”), II.A.4.a).(10) (“provide a learning and working environment in which residents have the opportunity to raise concerns and provide feedback”), and VI.B.6 (“provide a professional, equitable, respectful, and civil environment that is free from discrimination, sexual and other forms of harassment, mistreatment, abuse, or coercion”).^1^ In developing our goals and objectives in step 3, we aimed for participants to be able to define mistreatment and adapt strategies for addressing it directly with a communication tool we created. For step 4, when considering educational strategies, we modeled our workshop after similar modules in *MedEdPORTAL*, electing to include both didactics and case discussions in a single, hour-long workshop. This was followed by step 5, implementation, during which we implemented the workshop with two different audiences: once for residents at our institution and another time nationally with both learners and faculty. Finally, with step 6, evaluation and feedback, we created pre- and postactivity evaluations based on the session objectives and asked for comments on the material and cases.

Not only does this workshop raise awareness on the issue of resident mistreatment, it also provides resources and a tool with which residents can address any experienced mistreatment. The curriculum has been designed to be used with residents of any specialty and can be modified to include faculty, staff, and other learners as well. Although focused on individual resident experiences of mistreatment, this curriculum can be used as part of broader initiatives by institutions to address mistreatment within their learning environments.

## Methods

This workshop was developed by a team of the coauthors, inclusive of perspectives from a medical student, a resident, and a faculty member from two different institutions in the southwest United States. We delivered this workshop virtually at two different sessions in spring 2021, each spanning 60-90 minutes in length. The first workshop was delivered to 15 emergency medicine residents at their weekly afternoon conference at the University of New Mexico School of Medicine. The second was delivered at the Building the Next Generation of Academic Physicians conference to a national group of 25 students, residents, and faculty. In each session, we had two facilitators to colead the workshop and monitor the two small-group virtual breakout rooms. Institutional review board exempt status was granted by the University of New Mexico School of Medicine Human Research Protections Office, allowing for publication of the data obtained from our two implementations.

We designed a 60-minute interactive workshop. The first portion consisted of PowerPoint didactics to provide an overview of the research on resident mistreatment within the medical literature, focusing on the existing definition of mistreatment, the most common types of mistreatment, and the consequences of mistreatment (Appendix A). The second portion engaged participants in interactive case discussions and allowed them to practice using the communication tool for addressing mistreatment.

In order to construct a communication tool for residents to directly respond to mistreatment, we devised the acronym, REWIND (relax, express, why, inquire, negotiate, determine):

1. Relax and take a breath, take some time to collect your thoughts and avoid responding angrily.
2. Express what you heard, saw, or felt that was inappropriate.
3. Why was it inappropriate from your perspective?
4. Inquire about the other person's thoughts to hear their perspective and ensure that the conversation feels equal and productive.
5. Negotiate a more appropriate approach or response to the situation.
6. Determine a course of action moving forward to prevent future mistreatment.

This acronym was developed based on existing tools such as OWTFD, ORID (objective, reflective, interpretive, decisional), and GRIT (gather, restate, inquire, talk it out).^15,24,25^ In developing our acronym, we had several goals. First, we aimed to develop an acronym that would also be a mnemonic, to make it easier for participants to remember the steps of the communication tool. We appreciated the step-by-step framework presented by OWTFD but found its acronym difficult to remember. GRIT and ORID were easier to recall but lacked the specificity presented by OWTFD. With REWIND, we created an acronym that would be easier to remember and would be a step-by-step tool such as OWTFD. We included the basic steps laid out by the prior established communication tools (stating one's thoughts on the situation, asking for the other person's thoughts, and discussing how to proceed from the situation) but felt that it was important to also include the extra step of relaxing prior to engaging the situation of mistreatment.^15,24,25^ This allowed the resident to pause prior to engaging the other individual, as strong emotions could be invoked when experiencing mistreatment. The acronym was developed by the lead author (Joyce Pang), a senior resident and current education fellow in the University of New Mexico's Learning Environment Office, which focuses on addressing learner mistreatment at the institution. The acronym was also reviewed by other stakeholders in medical education, including medical students and faculty.

Centering on this acronym, we developed an interactive, case-based workshop via PowerPoint for residents, ideally to be presented during their junior years of training. In our implementations, the workshop was facilitated virtually by two of the coauthors, a senior resident and a medical student. The workshop could be facilitated in person or virtually and ideally would be led by a faculty member or upper-level resident. Our virtual implementation required only videoconferencing and email (for sending pre- and postworkshop surveys) capabilities. An in-person implementation would require a classroom, computer, and projector.

### Implementation

The time allotted for the workshop was 60 minutes. However, this could be extended to 90 minutes depending on participant group size and facilitator preference, to allow for longer case-discussion time and discussion of an additional case. Prior to the workshop beginning, we sent out a 5-minute preworkshop survey (Appendix B) to registrants. If the workshop is being implemented in person, we recommend that the preworkshop survey be given to participants as they enter the room. After personal introductions, we spent approximately 15 minutes on the didactic portion of the workshop. A sample timetable for different activities within the workshop was as follows:

• 5 minutes: welcome, introduction, presurvey.
• 15 minutes: didactics on mistreatment.
• 6 minutes: first case small-group discussion.
• 4 minutes: first case large-group discussion.
• 6 minutes: second case small-group discussion.
• 4 minutes: second case large-group discussion.
• 6 minutes: third case small-group discussion.
• 4 minutes: third case large-group discussion.
• 10 minutes: wrap-up, questions, postsurvey.

The PowerPoint slides began with an example case (case 1) of mistreatment, along with interactive polling software to assess the attendees’ initial perspectives. Participants were given an opportunity to provide comments regarding the first case. Introductory slides then discussed the definition and history of mistreatment in medical education, types of mistreatment, reasons for its prevalence, and general resources that could be used to address it. Next, 30 minutes were allotted to the case presentations and discussions. Four case scenarios (cases 2-5) were developed, highlighting different types of mistreatment and various interactions involving residents. These cases could be selected for presentation based on the preference of the facilitator. In our design, to fit within the constraints of resident education, we chose three of the four cases for presentation, with 10 minutes for each case. Six minutes were given for each small group, followed by 4 minutes for a large-group discussion for each case. The remaining 10 minutes of the session could be used to lengthen case discussions or to allow time for reflection on the activity. During the implementations, participants utilized this time to complete the postworkshop survey (Appendix C) before leaving.

### Facilitator Guide

The facilitator guide (Appendix D) included step-by-step instructions for the workshop and specific commentary for each slide. It also contained a breakdown for each of the five case scenarios to facilitate small-group discussions, as well as an explanation for each discussion question. Justification for each question was also provided to allow the facilitator to generate a robust discussion among participants.

### Evaluation

The pre- and postworkshop surveys (Appendices B and C) consisted of basic demographic questions, Likert-style questions assessing participants’ self-rated competency with the workshop objectives (Kirkpatrick level 1: reaction^26^), and free-form questions asking what participants liked or disliked about the workshop.^3^ We also included several optional questions to assess participants’ previous experiences with mistreatment. We compiled the survey responses from participants in both sessions in a deidentified, anonymous manner and analyzed them in aggregate. We compared the results of each matched question on the pre- and postworkshop surveys using a Wilcoxon signed rank test. Demographic data were analyzed using descriptive statistics, and thematic analysis was performed on responses to free-form questions.

## Results

The workshop was implemented virtually twice, first during a local resident education conference and second during a national medical education conference. There was a total of 40 participants, 17 of whom (42%) completed both the pre- and postworkshop surveys. Between the two implementations, among the participants who identified their roles, there were nine residents (53%), one physician-assistant resident (6%), one fellow (6%), two medical students (12%), and three faculty (18%). Among participants who reported their race and ethnicity, seven identified as Hispanic/Latino/of Spanish origin (42%), two identified as Black/African American (12%), and eight identified as White (47%). Nine of the participants were women (53%), and eight were men (47%).

On both the pre- and postworkshop surveys, participants were asked to self-assess their proficiency on six items related to the session objectives: the ability to (1) define mistreatment in medical education, (2) identify the resources for reporting mistreatment at their institution, (3) report mistreatment in medical education, (4) respond directly to mistreatment directed towards them, (5) respond directly to mistreatment they had observed, and (6) utilize the REWIND communication tool to respond to mistreatment. Participants were asked to rank their proficiency on a 5-point scale (1 = *not at all proficient*, 5 = *extremely proficient*). Because the workshop was directed towards empowering residents to address mistreatment, we isolated resident data (including that of the physician-assistant resident and fellow attendees, as both were also GME learners) to analyze the self-rated proficiency of the GME learners who participated (*n* = 11). Comparison of GME learner self-rated proficiency pre- and postworkshop using the Wilcoxon signed rank test demonstrated statistically significant improvement in the average rating for each question (see the Figure and Table).

**Figure.**
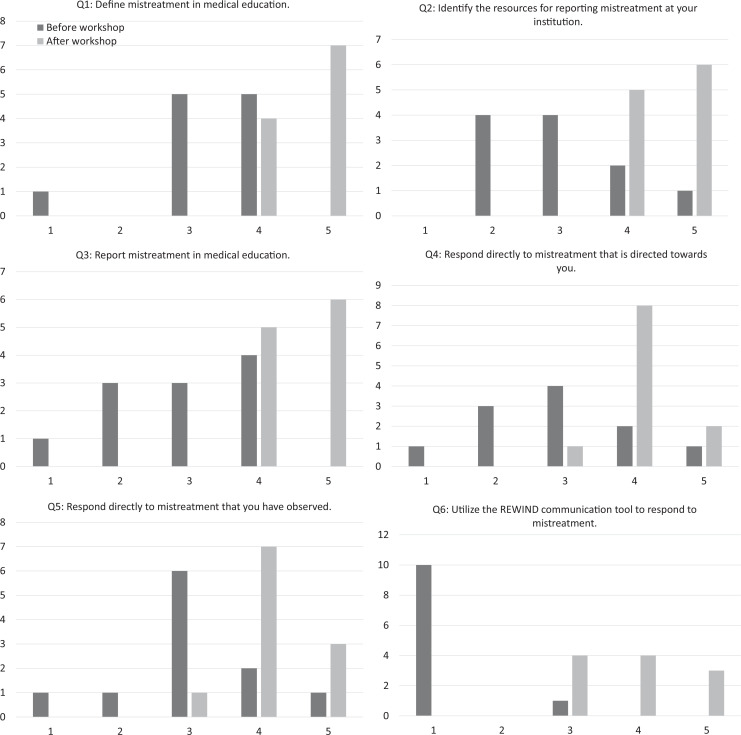
Number of workshop participants (*N* = 11) who self-rated proficiency on a 5-point Likert scale (1 = *not at all proficient*, 5 = *extremely proficient*) before and after the workshop. Abbreviation: REWIND, relax, express, why, inquire, negotiate, determine.

**Table.**
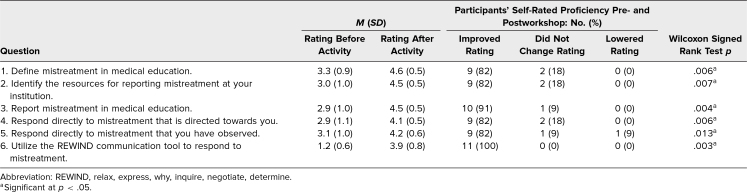
Self-Reported Proficiency Pre- and Postworkshop (*N* = 11)

Review of responses to the free-form questions, which asked what participants liked or disliked about the workshop, demonstrated two major groups of comments from GME learners. The first was that participants found the cases and small-group discussions particularly helpful, as demonstrated by the following participant quotes:

• “Cases were well written and had enough details to formulate an opinion.”
• “It addresses an important problem in medicine in a productive way. Educational and interactive. Effective method of delivering information. Great job.”
• “It was interactive and the cases were realistic.”
• “The scenarios, and different perspectives and ideas! Love the sharing of tools and ways to address and deal with issues.”

The second group of comments concerned the needed for more use of the REWIND tool during small-group discussions, as seen in the following quotes:

• “Would recommend having REWIND on the screen as small groups work through each case.”
• “More practice with the tool.”

The majority of comments were positive, highlighting points from the cases and discussions. One of the participants also noted that as an advanced practice provider (APP) resident, they were unsure how their experiences fit into the workshop and discussion.

## Discussion

This workshop uniquely focuses on the issue of resident mistreatment, teaching trainees about mistreatment and providing a tool with which to address mistreatment directly. This approach helps to fill the current gap in interventions on resident mistreatment. As institutions develop comprehensive plans for DEI, conversations and interventions around mistreatment will need to be initiated. Our workshop can be utilized as a part of this endeavor, as a method of introducing definitions of mistreatment, resources for reporting it, and how to address mistreatment of residents. Our implementations demonstrate that the workshop can increase GME learners’ self-rated proficiency on the session objectives. Participants’ reactions to the workshop have been positive, especially with regard to the core cases. This demonstrates the effectiveness and educational value of the workshop for residents.

However, we want to note several limitations of the workshop. The REWIND acronym was developed by the lead author (Joyce Pang), and although it was reviewed by other stakeholders (medical student, faculty), it did not go through a process of broad input or validation in development. Although the REWIND tool can be useful in many situations, it is limited by circumstances, including the user's comfort level and the target individual's demeanor and willingness to engage. Additionally, the tool is not a substitute for institutional efforts to prevent mistreatment and support learners who experience it. Second, the case presentations are not comprehensive and do not address all forms of mistreatment, and there is a degree of uncertainty and subjectivity within them that reflects the complexity of real-world situations. Because we evaluated workshop attendees at Kirkpatrick's level 1, reaction,^26^ we cannot draw conclusions about whether the workshop increased participants’ knowledge or skill or altered trainee or faculty behavior. Similarly, because the workshop was led by the same two presenters at our two implementations, we cannot reliably say whether similar results will be had with other presenters and audiences.

Next, we want to note some opportunities for improvement and further exploration. In the virtual setting, we had difficulties with making the REWIND tool available in breakout rooms. In response to this, we have created handouts outlining the cases and the REWIND tool that can be provided to participants prior to a virtual session or, if in person, during the session to help facilitate small-group discussion (Appendices E–I). We also want to note that our cases do not address all types of mistreatment. It is up to the facilitator to select those cases they feel are most pertinent to their institution. There is also flexibility for inclusion of cases unique to the institution, based on individual institutional reports or experiences.

To expand upon this work, future research should consider how REWIND and similar communication tools are utilized by workshop attendees. This consideration includes, but is not limited to, frequency of use, challenges to implementation, and overall impressions of effectiveness and ease of utilization. The REWIND tool can be adapted for specific departments or situations as needed and can be presented along with other training materials and institutional resources. The workshop can also be offered as part of a multifaceted institutional response to mistreatment, providing awareness and a tool for resident physicians. Future workshops could focus on the unique situations other trainees in the same environment face, including APP residents. Though we concentrate on residents in this workshop, it could also be helpful for other stakeholders in medical education, including faculty and medical students, which should be explored in future implementations. Ultimately, this work serves to fill a gap in the literature regarding the mistreatment of resident physicians and supplements existing educational material aimed at increasing awareness and addressing mistreatment in the clinical environment.

## Appendices


Mistreatment in Residency.pptxWorkshop Presurvey.docxWorkshop Postsurvey.docxFacilitator Guide.docxREWIND Handout.docxCase 2 Handout.docxCase 3 Handout.docxCase 4 Handout.docxCase 5 Handout.docx
*All appendices are peer reviewed as integral parts of the Original Publication.*
