# Supplementary material for: Mistreatment in Residency: Intervening With the REWIND Communication Tool
Source: MedEdPORTAL. 2022 Apr 26;18:11245. doi: 10.15766/mep_2374-8265.11245 (PMC9038987; doi:10.15766/mep_2374-8265.11245)
Supplement: Supplementary file 1 — Mistreatment in Residency.pptxWorkshop Presurvey.docxWorkshop Postsurvey.docxFacilitator Guide.docxREWIND Handout.docxCase 2 Handout.docxCase 3 Handout.docxCase 4 Handout.docxCase 5 Handout.docx [file mep_2374-8265.11245-s001.zip › C. Workshop Postsurvey.docx]

Mistreatment in Residency: Intervening with the REWIND Communication Tool: **Post-Workshop Evaluation**

**After** completing today’s workshop, how would you rate your ability to:

|  | **Not at all proficient** |  |  |  | **Extremely Proficient** |
| --- | --- | --- | --- | --- | --- |
| 1. Define mistreatment in medical education | 1 | 2 | 3 | 4 | 5 |
| 1. Identify the resources for reporting mistreatment at your institution | 1 | 2 | 3 | 4 | 5 |
| 1. Report mistreatment in medical education | 1 | 2 | 3 | 4 | 5 |
| 1. Respond directly to mistreatment that is directed towards **you** | 1 | 2 | 3 | 4 | 5 |
| 1. Respond directly to mistreatment that you have **observed** | 1 | 2 | 3 | 4 | 5 |
| 1. Utilize the REWIND communication tool to respond to mistreatment | 1 | 2 | 3 | 4 | 5 |

General Workshop Feedback:

|  | **Strongly Disagree** | **Disagree** | **Neutral** | **Agree** | **Strongly Agree** |
| --- | --- | --- | --- | --- | --- |
| 1. The topics covered in this workshop are relevant to my day-to-day experience. |  |  |  |  |  |
| 1. I would recommend this workshop to others. |  |  |  |  |  |
| 1. The cases were relevant. |  |  |  |  |  |
| 1. The cases were realistic. |  |  |  |  |  |
| 1. I enjoyed the workshop. |  |  |  |  |  |

What did you like about the workshop? _________________________________________________________________

__________________________________________________________________________________________________

What did you dislike about the workshop? _______________________________________________________________

__________________________________________________________________________________________________

Recommendations for future workshops: ________________________________________________________________

__________________________________________________________________________________________________

Additional comments: _______________________________________________________________________________

__________________________________________________________________________________________________
