# Supplementary material for: Mistreatment in Residency: Intervening With the REWIND Communication Tool
Source: MedEdPORTAL. 2022 Apr 26;18:11245. doi: 10.15766/mep_2374-8265.11245 (PMC9038987; doi:10.15766/mep_2374-8265.11245)
Supplement: Supplementary file 1 — Mistreatment in Residency.pptxWorkshop Presurvey.docxWorkshop Postsurvey.docxFacilitator Guide.docxREWIND Handout.docxCase 2 Handout.docxCase 3 Handout.docxCase 4 Handout.docxCase 5 Handout.docx [file mep_2374-8265.11245-s001.zip › D. Facilitator Guide.docx]

**Mistreatment in Residency: Intervening with the REWIND Communication Tool**

**Facilitator Guide**

Joyce Pang MD, Natasha Navejar BS, John Paul Sánchez MD, MPH

**Workshop Overview**

To address resident mistreatment, we present an interactive workshop for residents to learn about mistreatment in the clinical environment and introduce REWIND, a step-by-step communication tool for responding to perceived incidents. After a brief overview of mistreatment, up to five cases may be presented followed by discussions about the nature of the interactions. We recommend that three cases be chosen based on institutional preference to stay within a one-hour time block. We determined that four out of the five cases are mistreatment, with the exception being a common misconception of mistreatment, namely feedback. We have provided questions to trigger discussion about the potential mistreatment within each case. The workshop is amenable to a virtual or an in-person format, with attached PowerPoint including introduction, background, cases with discussion questions, and pre- and post-surveys regarding resident attitudes toward the material herein.

**Learning Objectives**

1. Define the most common forms of resident physician mistreatment.

2. Demonstrate the use of the REWIND communication tool to address mistreatment.

3. Adapt strategies to address experienced or observed mistreatment.

**PowerPoint Guide**

We encourage facilitators to modify the PowerPoint as needed to reflect current research as well as the clinical environment in which participants are practicing. Only one facilitator is necessary for the PowerPoint presentation, but more may be useful for the small-group case discussions (see Slide 19).

**Slide 1:** Title Slide. Add names, degrees and positions, and institutional information of facilitators to slide. Introductions should commence when this slide is presented. Facilitators may cite this peer-reviewed module.

**Slide 2:** Any and all relevant financial disclosures or conflicts of interest should be listed here.

**Slide 3:** Announce the learning objectives as listed on this slide (same as above).

**Slide 4:** We recommend reading off an example case, Maria’s Case #0, to introduce participants to the case-based format later in the presentation and to encourage early engagement.

**Slide 5:** Ask the participants in the large group if they think that the presented case is mistreatment, either verbally or with polling software. We recommend encouraging participants to also share their reasoning with everyone, which will surely tie into some of the types of mistreatment discussed later. Refer to the Case Guide below for a summary and assessment of this case that may be used to guide discussion.

**Slide 6:** Read off the AAMC definition of mistreatment and the ACGME guidelines, which mandate that programs must provide an environment free from mistreatment along with education and resources for identifying and addressing it. Facilitators may choose to specifically emphasize the bold yellow text to highlight the take-aways from these quotations.

**Slide 7:** History of mistreatment in medical education. This slide primarily focuses on medical student mistreatment, as does the majority of research literature. Read off bullet points. This may be compared and contrasted with the information on resident mistreatment in the next slide.

**Slide 8:** This slide cites some of the limited literature on the prevalence of resident physician mistreatment. Facilitators may read these statistics off of the slide, and add their own data if available.

**Slide 9:** A list of the types of mistreatment. Read off the list, noting that the types in bold yellow are some of the more pervasive in the clinical environment.

**Slide 10:** Think back on Maria’s case--what types of mistreatment do you recognize?

Allow participants to say aloud some of the types of mistreatment they recognized from Maria’s case before revealing the answers: verbal abuse, public humiliation, neglected and/or left out, mistreated based on gender or gender identity. Communicate that like in real life, not all cases of mistreatment will be clear-cut; for instance, in this case, it is unclear if the attending may have been treating Maria harshly due to her gender.

**Slide 11:** This slide discusses why mistreatment may occur, with suggested hypotheses like the cultural hierarchy of medicine, the misconception that it may help trainees learn, the gendered nature of the profession, and generational differences including an increasingly diverse population of trainees and changes in training guidelines. Read off the bullet points, and bring attention to the pie graph on the right-hand side of the slide, which shows the diverse racial composition of 2018-2019 U.S. medical school graduates.

**Slide 12:** This slide emphasizes the deleterious consequences of resident physician mistreatment. Read the statistics off the bullet points.

**Slide 13:** This slide shows the ACGME common program requirements, and has a bullet point remarking on how addressing mistreatment is necessary for continued accreditation. Facilitators may bring up how this may deter residents from reporting, as their own reports may affect their program’s accreditation and inadvertently threaten their ability to become board-certified.

**Slide 14:** What can be done about mistreatment? Read off the three possibilities listed on the slide.

**Slide 15:** Resources at your institution. This slide offers some general resources that may be useful at any institution, but is ultimately meant to be edited to feature relevant institution-specific resources.

**Slide 16:** How do I address it in the moment? This is a transition slide we use to introduce the REWIND communication tool.

**Slide 17:** What is REWIND? Read off the acronym and its associated meaning. We recommend that a printout of the REWIND tool be physically passed out to participants if in-person (virtually sent otherwise). This will help participants remember and implement the tool for the upcoming case discussions.

**Slide 18:** When is REWIND best used? List off our suggestions for optimal use.

**Slide 19:** Going back to Maria’s case, this slide illustrates a use of REWIND to address her situation.

**Slide 20:** Transition to breakout rooms/small groups. The number of facilitators and small groups may vary depending on time constraints, desired number of cases, and number of participants. One facilitator may suffice in an in-person setting, but we recommend at least two facilitators if breaking out into virtual small groups in order to monitor and promote virtual discussion. With our workshops being virtual and having between 10-20 participants, we decided to split up into two groups with a facilitator in each to guide participants through the case discussion questions.

**Case Guide**

We recommend using the questions listed after each case in the PowerPoint to facilitate discussion. Each case should take approximately 10 minutes to read and discuss in large- or small-groups. Below are the summaries for the cases, each with an assessment that discusses the judgment of the case (i.e. mistreatment or not mistreatment), possible obstacles to reporting, and suggested implementation of the REWIND communication tool. If desired, participants may roleplay the scenario and practice use of the REWIND communication tool to respond to the incident. Afterward, they can compare their responses to our provided examples. These REWIND response examples are meant to be a guide, and we strongly encourage the participants to come up with their own responses during the case discussions. The case handouts are available for distribution to each of the small groups, so that they have a reference to the case and discussion questions during the small group discussion.

**Case #1 Operation Room Rage**

**Summary:** A female resident, Maria, accidentally drops a surgical instrument in the operating room, which triggers her male attending Dr. Lansing to yell an expletive before cutting her apology off abruptly. He later gives an opportunity to tighten one of the last orthopedic bolts to the male medical student, with a snide remark about trusting him to finish the job.

**Assessment:** This case is an example of mistreatment, not only for the unprofessionalism in the attending cursing, but also his subtle remark hinting that he cannot trust Maria in the operating room. While Maria’s mistake may delay the patient’s surgery, it does not warrant punishment, humiliation, or gender-based discrimination. Dr. Lansing’s behavior therefore is unacceptable, and his last remark may be understandably hurtful to a female trainee in a male-dominated field. In this scenario, it is understandable that Maria may elect not to use REWIND at all, considering Dr. Lansing’s temper in the operating room and the fact that he already curtailed her initial attempt at communication.

**REWIND example:**

**R**: Maria may pause after the case ends to collect herself, determine what she wishes to say, and identify an appropriate time and place to meet with Dr. Lansing (e.g. his office after the cases are done for the day).

**E**: Maria: “Dr. Lansing, I wanted to meet with you to debrief about what happened in the OR today. I made a mistake by dropping one of our equipment pieces, but I was taken aback by your response. I was not expecting to be shouted at or for my apology to be cut off. I was hoping to be involved with the case more, but I felt that after my mistake, you put more trust in the medical student than in me.”

**W**: Maria: “I found the situation hurtful because I made an honest mistake, and I wanted nothing more than to help make it right. I know that although it was an accident, I may have cost the patient and the staff unnecessary time in the OR and I do apologize for that and wish it never happened. But I felt that the way you responded was unfair to me as a learner.”

**I**: Maria: “With that being said, I want to hear your thoughts because I believe that we can have better communication moving forward.”

Dr. Lansing: “Maria, I do feel bad about what I said earlier. I don’t want you to take it personally because I was having a rough day today, and I let my emotions get the best of me. I apologize.”

**N**: Maria: “Thank you for saying that, Dr. Lansing. I just don’t want to be put in that uncomfortable situation again.”

Dr. Lansing: “That is completely reasonable, Maria. You shouldn’t have to be.”

**D**: Maria: “Great. Well, I would like to start fresh tomorrow because I know I have a lot to learn from you and gaining more autonomy in the OR is my current goal. How does that sound?”

Dr. Lansing: “Absolutely, Maria. Moving forward I intend to be more mindful of my actions in the operating room.”

**Case #2 Patient Overload**

**Summary:** A Spanish-speaking resident, Eduardo, receives a heavier patient load than his peers and is assigned patients based on language without consideration for his learning goals.

**Assessment:** We would define the scenario presented in this case as mistreatment, as it constitutes a discriminatory act that results in Eduardo receiving a disproportionately heavy workload with disregard to his learning goals. It also prevents other trainees from learning cultural competence, using an interpreter, or practicing Spanish. Because this may not be an intentional offense by Mark, it is potentially correctable with use of the REWIND communication tool alone. If Mark is unreceptive to Eduardo’s request for equitable treatment, Eduardo may need to report the mistreatment to leadership at his program. Eduardo may be hindered in reporting this offense due to a fear of retaliation or self-doubt about whether his situation constitutes mistreatment. Other potential reasons include the emotional stress that would be associated with reporting and the desire to still provide excellent care for his Spanish-speaking patients.

**REWIND example:**

**R**: Eduardo may need some time to collect his thoughts and take care of any immediate patient care duties before finding a time and private space to meet with Mark.

**E**: Eduardo: “Thanks for taking a minute to talk with me, Mark. I wanted to bring up something that has been bothering me recently. I have noticed that the patients assigned to me tend to all be Spanish-speaking. I also have double the number of patients as the other residents on the team at the moment.”

**W**: Eduardo: “While I do appreciate being able to give those patients care in their own language, I feel like it is starting to come at the cost of my learning goals. I was hoping to have patients with a variety of pathologies, but if a Spanish-speaking patient arrives, I feel that I am expected to take them even if I have already managed their illness many times over. The extra workload is also becoming overwhelming.”

**I**: Eduardo: “I know this all may be a lot to take in, and I want to hear your thoughts.”

Mark: “Eduardo, I’m sorry that you’ve been feeling this way. I did not mean to overwhelm you with patients, especially those that you may not have preferred to see. I was trying to prioritize an efficient workflow and thought that I was doing right by the patients by giving them a Spanish-speaking physician. But now that you are telling me all of this, I am realizing that I wasn’t thinking about your wellbeing or learning.”

**N**: Eduardo: “I understand, Mark. How do you feel about dividing the patients more equitably among the residents? It would also be an opportunity for my co-residents to work more with Spanish-speaking patients. And I’d like to see the hepatitis patient instead of the COPD patient, too, if that’s okay.”

Mark: “Absolutely, Eduardo. I’m sorry again.”

**D**: Eduardo: “It is alright. We can be more communicative in the future to make sure we are on the same page, okay?”

Mark: “Definitely. I’ll ask for your input and listen to any concerns you have. Please don’t hesitate to speak up again if something is bothering you.”

**Case #3 Outed**

**Summary:** An LGBTQ-identifying resident’s sexual orientation is casually disclosed by an attending to a female patient as a way of offering reassurance about having a man conduct her pelvic exam.

**Assessment:** We would define this situation as mistreatment because a resident’s sexual orientation is inappropriately shared with a patient. This is not only unprofessional, but also discriminatory to disclose such private information about an LGBTQ-identifying individual. While Dr. Silva may have had the intention of reassuring the patient, her behavior is unacceptable. REWIND may be used here by Brayden to address the mistreatment with Dr. Silva or another member of program leadership to ensure that it does not happen again. The incident also makes inappropriate assumptions about both Brayden and the patient, who may have pertinent reasons for not wanting a male OB-GYN that may warrant further inquiry. Brayden may be hesitant to report this incident since the details of the situation are inherently tied to his sexual orientation, which he may fear being shared with more people. He also may not think Dr. Silva’s actions constituted mistreatment, since it was said casually and likely without ill intent.

**REWIND example:**

**R**: Brayden may need some time to reflect after such an unexpected and inappropriate remark by Dr. Silva. If Brayden is able, he should try to address the incident privately with her as soon as he is finished with the patient encounter.

**E**: Brayden: “Hi Dr. Silva, thanks for taking a minute to talk. I felt like I needed to discuss something you said earlier. I think you may have been trying to make a joke or reassure our last patient, but you made a comment that I didn’t “bat” for her team.”

**W**: Brayden: “The comment caught me off-guard, as I wasn’t expecting my sexual orientation to be addressed in front of a patient like that. It was inappropriate, although you may have had good intentions.”

**I**: Brayden: “I want to hear your thoughts about it.”

Dr. Silva: “I am feeling very ashamed right now. I have had a great working relationship with you before this, and I guess I was trying to make a casual joke, but it was inappropriate. I immediately realized it was unprofessional and wanted to take it back. I probably made the patient very uncomfortable, too. Instead of making that comment, I could have inquired about her obvious discomfort surrounding the pelvic exam.”

**N**: Brayden: “I forgive you, Dr. Silva, and I suspected you didn’t have bad intentions. I just wanted to let you know that I did not want that kind of personal information discussed at work again.”

**D**: Brayden: “Does that sound like a plan to you, Dr. Silva?”

Dr. Silva: “Absolutely. I truly apologize again and promise to be more professional.”

**Case #4 “Stupid” Questions**

**Summary:** Rather than embracing a teaching moment, an attending responds with condescension (“Stop asking stupid questions”) when asked by a resident about the appropriate management of a patient.

**Assessment:** This is a clear instance of mistreatment. Dr. Brown is overtly and undeservingly rude to Grace, who is seeking instruction from him. Dr. Brown could have respectfully addressed her question and given proper reasoning for why he wanted to continue drawing labs on Mr. Fiori. Grace can attempt to employ the REWIND communication tool, but it is very possible that Dr. Brown could be an unwilling participant. Thus, she should also prepare to report the incident to program leadership, possibly with the support of the other trainees who have witnessed or experienced Dr. Brown’s unprofessionalism first-hand. Grace may be hesitant to do this due to fear of retaliation by Dr. Brown, emotional stress from reporting, or the self-doubt about whether the incident is mistreatment (i.e. tolerating abrasive personalities is part of the job).

**REWIND example:**

**R**: Grace may need to muster courage, let her fear or anger quell, and gather her thoughts before taking Dr. Brown aside for a discussion.

**E:** Grace: “Dr. Brown, thanks for taking a minute to talk. I just wanted to address something you said earlier when I asked about discontinuing labs on our patient. You told me to stop asking stupid questions and threatened the possibility of a lawsuit.”

**W**: Grace: “I asked my question because I generally wanted to learn from you and hear your clinical approach to decision-making. I was hurt by your response, and it has made me hesitant to ask you more questions even though I am trying to learn.”

**I**: Grace: “What are your thoughts on this?”

Dr. Brown: “My thoughts on this? Use your brain, those are my thoughts! Are they not teaching you this in medical school?!”

**N**: Grace: “You don’t think that your teaching style could use any improvement, Dr. Brown?”

Dr. Brown: “I’ve been teaching for longer than you have been alive.”

**D**: Grace: “Okay, I see. Well, I am sorry you feel that way. I kept the labs on like you asked. I was just hoping to learn why from you.”

Dr. Brown: “Sorry to disappoint you then.”

In this particular example, the attending has a negative response to the resident who is attempting to communicate with the REWIND tool. We wanted to provide this as an example of a situation in which mistreatment continues in further communication, and a situation which should be escalated to program leadership. This may include the program director, department chair, office of graduate medical education, and/or other institutional organizations. The facilitator can use this as an opportunity to re-emphasize the resources available for residents at the individual institution.

**Case #5 Running Late**

**Summary:** A resident, Shawna, is running late for work and asks a newly minted clinical medical student to chart review and gather numbers for her patients. The medical student, new to the service and to clinical rotations, cannot effectively carry out Shawna’s duty, leaving her team to slowly do the task in her absence. The attending, Dr. Pond, explains to Shawna why her behavior is inappropriate in the call room with the other residents, leaving Shawna feeling ashamed and later upset toward Dr. Pond for the public criticism.

**Assessment:** We do not consider this situation mistreatment as Dr. Pond respectfully addressed Shawna’s tardiness and discussed why her behavior was inappropriate. While the medical student has a responsibility to assist the medical team, he was not properly trained to complete the work asked of him. This led to delays in chart reviewing that may impact patient care. While Shawna may be upset that her feedback was given in front of the medical student and the team, the consequences of her mistake were clear to her colleagues, who are finishing her work. Dr. Pond appropriately takes this opportunity to provide a teaching moment to Shawna and reassure the medical student, who is likely experiencing his own sense of shame for not being able to carry out Shawna’s task. Shawna may still use the REWIND communication tool with Dr. Pond. This may result in a mutual understanding between the two, with Shawna gaining an appreciation for feedback and Dr. Pond setting expectations for the future.

**REWIND example:**

**R**: Shawna may need some time to process the situation that happened that morning and find a reasonable time afterward to pull Dr. Pond aside for a private discussion.

**E**: Shawna: “Thanks for meeting with me, Dr. Pond. I just wanted to talk about what happened this morning. I truly apologize for running late this morning and giving the new medical student a task he was not adequately prepared for. I know now that that must have put him in an uncomfortable position. And my team had to step away from their own work to help him through it. But I also want to address the manner in which you gave me my feedback. It was said in front of the whole team, including the medical student.”

**W**: Shawna: “While what you said may have been true, I felt that saying it in front of the entire team may have unnecessarily hurt my esteem and embarrassed me.”

**I**: Shawna: “I’d like to hear your thoughts on the situation.”

Dr. Pond: “Shawna, I appreciate you bringing this up. I promise that I had no intention of embarrassing you, but I felt that I needed to speak up to set clear expectations for you, the medical student, and the rest of the team. Your actions had clear consequences that were known to the team, so I did not feel like I was out of bounds in doing so. As a part of team-based learning, I want there to be a safe space for group feedback and response.”

Shawna: “I hadn’t thought about it that way and I do understand that feedback is an important aspect to my training.”

**N**: Shawna: “How do you think we should proceed from here, Dr. Pond?”

Dr. Pond: “Let’s meet with the team to discuss group feedback and talk about team member communication and responsibilities.”

**D:** Shawna: “I understand, and I do appreciate the feedback.”
