# Supplementary material for: Mistreatment in Residency: Intervening With the REWIND Communication Tool
Source: MedEdPORTAL. 2022 Apr 26;18:11245. doi: 10.15766/mep_2374-8265.11245 (PMC9038987; doi:10.15766/mep_2374-8265.11245)
Supplement: Supplementary file 1 — Mistreatment in Residency.pptxWorkshop Presurvey.docxWorkshop Postsurvey.docxFacilitator Guide.docxREWIND Handout.docxCase 2 Handout.docxCase 3 Handout.docxCase 4 Handout.docxCase 5 Handout.docx [file mep_2374-8265.11245-s001.zip › E. REWIND Handout.docx]

Mistreatment in Residency: An Overview and Intervening with the REWIND Communication Tool

**Handout: REWIND Communication Tool**

| **What is REWIND?** |
| --- |

- **REWIND** is a communication tool that can be used to address mistreatment in the moment

| **When is REWIND best used?** |
| --- |

- When the person you want to address is **open to a line of communication and amenable to reflecting on their behavior** (and potentially changing it!)
- When the **behavior you’re experiencing is identifiable and communicable**
- When you are **calm and able to speak with composure**
- When you feel **safe**
- When addressing a **microaggression**

| **What is REWIND?** |
| --- |

**Relax** - Take a breath, collect your thoughts. Do not respond with anger or frustration.

**Express** - Express what the statement you heard was.

**Why** - Why was the statement hurtful or inappropriate?

**Inquire** - Inquire upon the other person’s thoughts. Give them a chance to speak.

**Negotiate** - Discuss what may be a better approach.

**Determine** - Determine how to act in the future.
