# Supplementary material for: Mistreatment in Residency: Intervening With the REWIND Communication Tool
Source: MedEdPORTAL. 2022 Apr 26;18:11245. doi: 10.15766/mep_2374-8265.11245 (PMC9038987; doi:10.15766/mep_2374-8265.11245)
Supplement: Supplementary file 1 — Mistreatment in Residency.pptxWorkshop Presurvey.docxWorkshop Postsurvey.docxFacilitator Guide.docxREWIND Handout.docxCase 2 Handout.docxCase 3 Handout.docxCase 4 Handout.docxCase 5 Handout.docx [file mep_2374-8265.11245-s001.zip › F. Case 2 Handout.docx]

Mistreatment in Residency: An Overview and Intervening with the REWIND Communication Tool

**Handout: Case #2**

| **Case #2** |
| --- |

Eduardo is an intern in Internal Medicine on the hospitalist service at the local county hospital, which serves a high volume of underserved patients who are predominantly Hispanic. Eduardo, a native Spanish-speaker, rotated here frequently as a medical student, where he readily offered his interpreter skills if the need arose.

While Eduardo takes pride in leveraging his bilingualism to help Spanish-speaking patients understand their medical care, he is starting to notice that his language competency is affecting his patient load.

“Hey Eduardo, do you mind taking Suarez?” asks Eduardo’s chief resident, Mark.

Eduardo looks over at the markerboard in the office, which shows his patient list already doubling his colleagues’ lists. “Well, I was actually thinking about taking Smith if anyone, since I haven’t managed a patient with hepatitis yet.”

“I think Suarez will be a good learning opportunity as a COPD patient and you will be able to get through the interview in the amount of time it takes the other residents to find an interpreter,” Mark explains.

Eduardo agrees to oblige, but turns back to his computer feeling overwhelmed by his current workload and thinking about how he has already overseen the care of five COPD patients during this rotation, all of whom were Spanish-speakers. He has tried to remain optimistic about the situation, but cannot help but feel like his workload compared to his peers is disproportionately heavy with less consideration for his learning goals.

| **Case #2 Discussion** |
| --- |

Is this mistreatment or is Eduardo just being a “team player”?

How can Eduardo address it with his chief resident? Can the REWIND communication tool be used?

What are other avenues Eduardo can use to address this if his chief resident does not respond?

What are potential reasons that may prevent Eduardo from reporting this as mistreatment?

Practice how Eduardo might use REWIND in this situation.
