# Supplementary material for: Mistreatment in Residency: Intervening With the REWIND Communication Tool
Source: MedEdPORTAL. 2022 Apr 26;18:11245. doi: 10.15766/mep_2374-8265.11245 (PMC9038987; doi:10.15766/mep_2374-8265.11245)
Supplement: Supplementary file 1 — Mistreatment in Residency.pptxWorkshop Presurvey.docxWorkshop Postsurvey.docxFacilitator Guide.docxREWIND Handout.docxCase 2 Handout.docxCase 3 Handout.docxCase 4 Handout.docxCase 5 Handout.docx [file mep_2374-8265.11245-s001.zip › G. Case 3 Handout.docx]

Mistreatment in Residency: An Overview and Intervening with the REWIND Communication Tool

**Handout: Case #3**

| **Case #3** |
| --- |

Brayden Park is an OB/GYN intern stepping into a patient’s room along with his attending, Dr. Silva, who is observing Brayden’s clinical history-taking and physical exam skills.

“Hi Mrs. Rogers, I am Dr. Silva, the attending physician. And this is Dr. Park, who will be overseeing your care,” she explains, gesturing for Brayden to take over the encounter.

After exchanging pleasantries and gathering a thorough history on Mrs. Roger’s abnormal uterine bleeding, Brayden explains that he will be doing her pelvic exam next.

The patient, already dressed in a blue paper gown, furrows her brow and seems to shift nervously on the exam table.

“Is that alright with you?” Brayden asks, taking note of the patient’s sudden discomfort.

“Oh well, it’s fine I suppose. I just thought a woman doctor would be the one doing it,” replies Mrs. Rogers.

“Oh honey, if it makes you feel better, he doesn’t even bat for your team,” chimes Dr. Silva.

Brayden’s face grows red and hot. Although flustered, he maintains his composure and continues with the encounter after getting consent from the patient to proceed.

| **Case #3 Discussion** |
| --- |

Is this mistreatment? Why or why not?

How can Brayden address it with his attending? Can the REWIND communication tool be used?

What are other avenues Brayden can use to address this if his attending does not respond?

What are potential reasons that may prevent Brayden from reporting this as mistreatment?

Practice how Brayden might use REWIND in this situation
