# Supplementary material for: Mistreatment in Residency: Intervening With the REWIND Communication Tool
Source: MedEdPORTAL. 2022 Apr 26;18:11245. doi: 10.15766/mep_2374-8265.11245 (PMC9038987; doi:10.15766/mep_2374-8265.11245)
Supplement: Supplementary file 1 — Mistreatment in Residency.pptxWorkshop Presurvey.docxWorkshop Postsurvey.docxFacilitator Guide.docxREWIND Handout.docxCase 2 Handout.docxCase 3 Handout.docxCase 4 Handout.docxCase 5 Handout.docx [file mep_2374-8265.11245-s001.zip › H. Case 4 Handout.docx]

Mistreatment in Residency: An Overview and Intervening with the REWIND Communication Tool

**Handout: Case #4**

| **Case #4** |
| --- |

Grace is an intern on her third week of an internal medicine rotation. She has heard that she will have a new attending this week, Dr. Brown. Her colleagues seem apprehensive about him based on rumors they have heard about his “abrasive” teaching style.

“I heard he made Sarah cry last week by chewing her out for not getting a patient transferred to the neurology service fast enough,” mutters John, a fellow intern.

Grace is troubled by these rumors, but decides not to form any judgments on Dr. Brown just yet. *Maybe he was just having a bad day.* Minutes later, Dr. Brown arrives. After introductions are made, Grace inquires about the management of one of her patients.

“Dr. Brown, for Mr. Fiori, is it alright to discontinue his daily CBC and BMP? He has been clinically stable and there is no indication I am aware of. He should be leaving in a couple of days once his housing situation is taken care of.”

“And if he crashes tomorrow, then what?”

“Well, I—”

“Are you going to handle the lawsuit?”

“I just thought that conserving hospital resources—”

“I didn’t think so. Keep the labs on and stop asking stupid questions.” Grace turns back to her computer dutifully while her colleagues exchange troubled side glances with one another.

| **Case #4 Discussion** |
| --- |

How could Grace’s question have been better addressed?

Can the REWIND communication tool be used here to discuss the incident with Dr. Brown? If not, what other options does Grace have?

What are potential reasons that may prevent Grace from reporting this as mistreatment?

Practice how Grace might use REWIND in this situation
