# Supplementary material for: Mistreatment in Residency: Intervening With the REWIND Communication Tool
Source: MedEdPORTAL. 2022 Apr 26;18:11245. doi: 10.15766/mep_2374-8265.11245 (PMC9038987; doi:10.15766/mep_2374-8265.11245)
Supplement: Supplementary file 1 — Mistreatment in Residency.pptxWorkshop Presurvey.docxWorkshop Postsurvey.docxFacilitator Guide.docxREWIND Handout.docxCase 2 Handout.docxCase 3 Handout.docxCase 4 Handout.docxCase 5 Handout.docx [file mep_2374-8265.11245-s001.zip › I. Case 5 Handout.docx]

Mistreatment in Residency: An Overview and Intervening with the REWIND Communication Tool

**Handout: Case #5**

| **Case #5** |
| --- |

Shawna is an intern on her surgery rotation at a large pediatric hospital. As the intern, she is tasked with arriving early to the hospital each morning to review patient charts in preparation for 5:30AM rounding with the rest of the team. This morning, Shawna is running late.

Realizing that she will not have enough time to gather the pertinent information from the chart on all the patients, she decides to text the medical student, Josh, who is starting on the service today: “Running late. Can you gather the numbers for the patients today? Thank you so much!!”

When Shawna arrives to the call room, her team members are all on the computers instead of gathered around the conference table discussing patients as they usually are.

“Hey guys, sorry I am late. The medical student got the numbers, though, right?”

No one replies, and Shawna realizes the other residents are all busy chart reviewing and printing out the patient lists—tasks that she typically would have completed by this time. A dark-haired boy in scrubs approaches Shawn sheepishly.

“Hey Shawna, I’m Josh, the medical student. I received your text and tried to chart review as best as I could, but I couldn’t do it as fast as you probably could because I’m unfamiliar with the charting system. I’m sorry.”

The attending, Dr. Pond, rises from his chair in the corner of the room.

“It’s not your fault, Josh,” says Dr. Pond. He then turns his attention to Shawna. “Shawna, I know accidents happen. But the numbers were your responsibility. Josh can help you in the future, but it was not fair of you to ask a medical student on his first day here—and on his first rotation ever, no less—to gather all the pertinent information from the EMR without training him first.”

Shawna nods silently, and mutters an apology. While she feels ashamed in the moment, she later starts to feel slighted that the attending would call her out on her mistakes in front of her colleagues and the medical student.

| **Case #5 Discussion** |
| --- |

Is this mistreatment (e.g. public humiliation) or feedback?

Do you think the attending handled this situation well? If not, what could Dr. Pond have done better?

How should Shawna and the attending discuss this further if she is concerned? Can the REWIND tool be used?

Practice how Shawna might use REWIND in this situation.
