# Supplementary material for: Mistreatment in Residency: Intervening With the REWIND Communication Tool
Source: MedEdPORTAL. 2022 Apr 26;18:11245. doi: 10.15766/mep_2374-8265.11245 (PMC9038987; doi:10.15766/mep_2374-8265.11245)
Supplement: Supplementary file 1 — Mistreatment in Residency.pptxWorkshop Presurvey.docxWorkshop Postsurvey.docxFacilitator Guide.docxREWIND Handout.docxCase 2 Handout.docxCase 3 Handout.docxCase 4 Handout.docxCase 5 Handout.docx [file mep_2374-8265.11245-s001.zip › A. Mistreatment in Residency.pptx]

## Slide 1
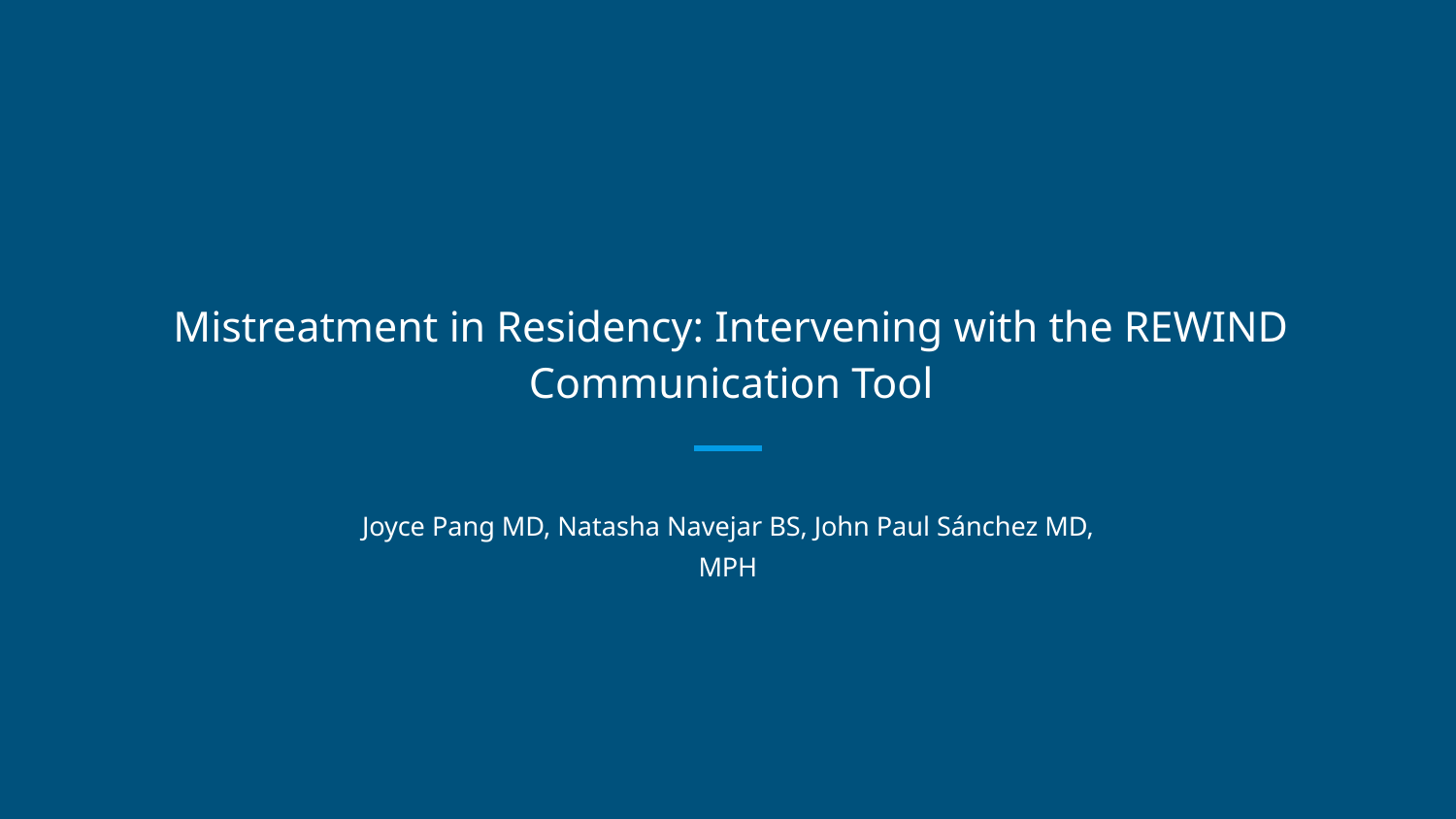

# Mistreatment in Residency: Intervening with the REWIND Communication Tool
Joyce Pang MD, Natasha Navejar BS, John Paul Sánchez MD, MPH

## Slide 2
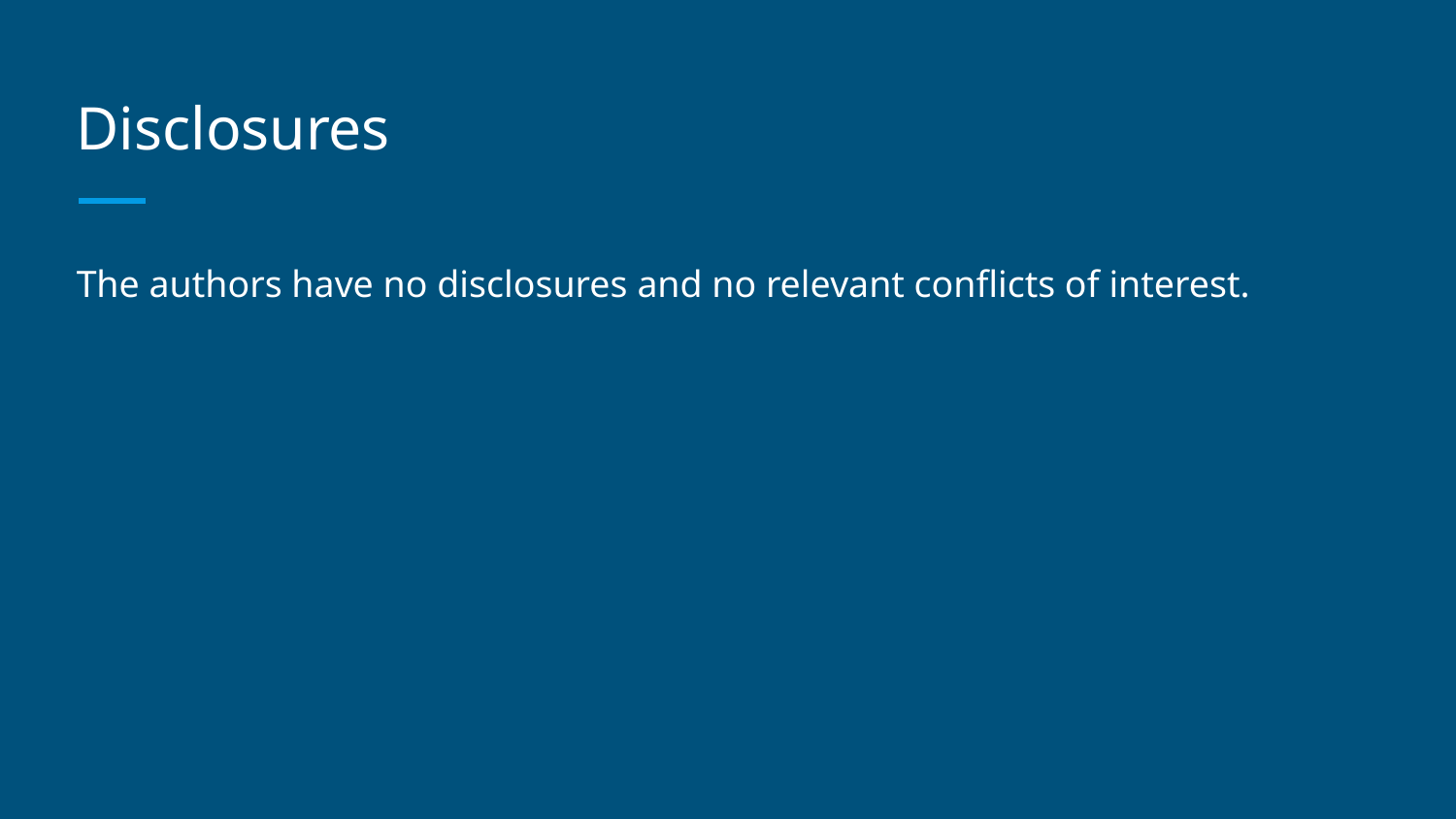

# Disclosures
The authors have no disclosures and no relevant conflicts of interest.

## Slide 3
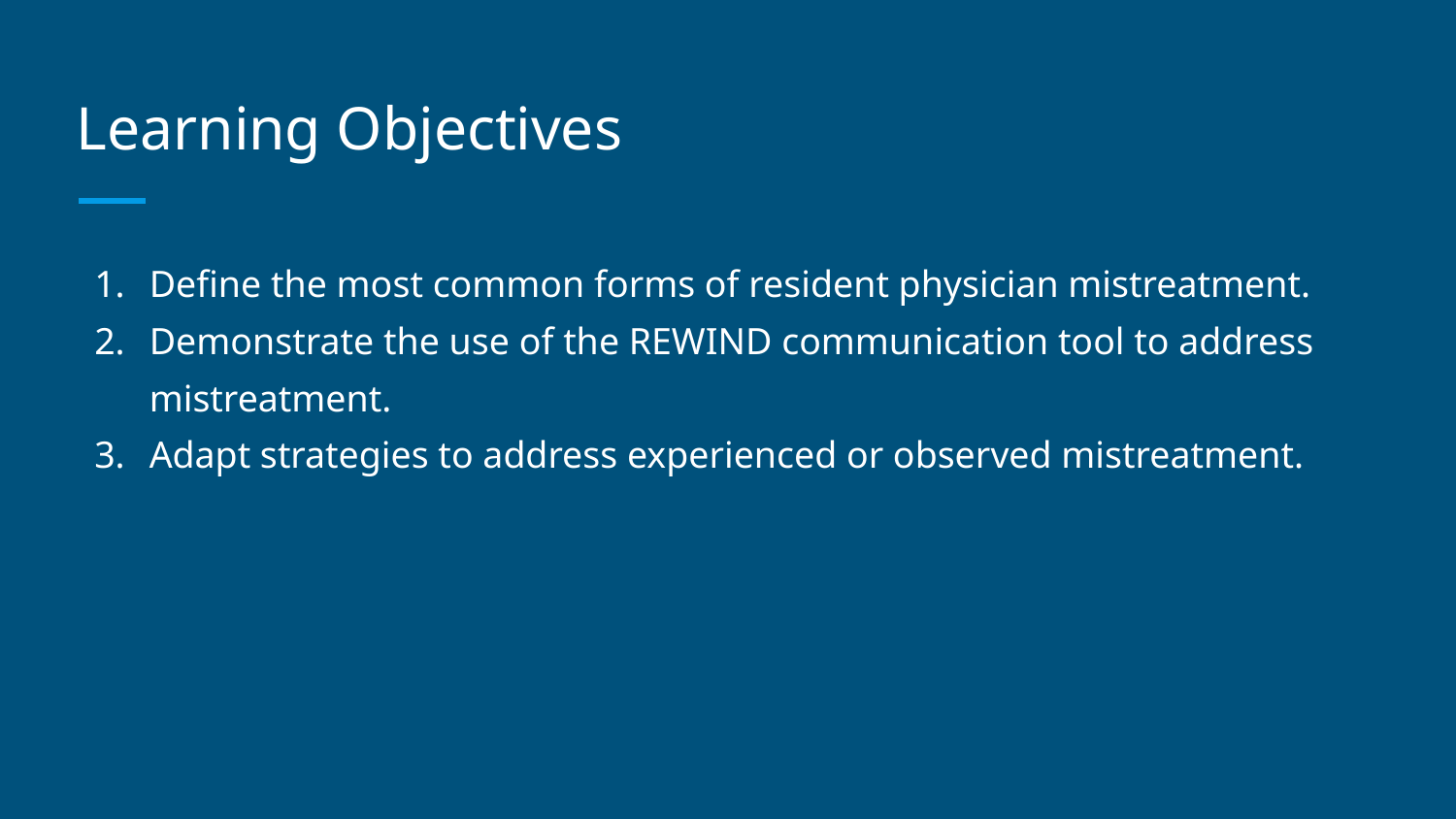

# Learning Objectives
Define the most common forms of resident physician mistreatment.
Demonstrate the use of the REWIND communication tool to address mistreatment.
Adapt strategies to address experienced or observed mistreatment.

## Slide 4
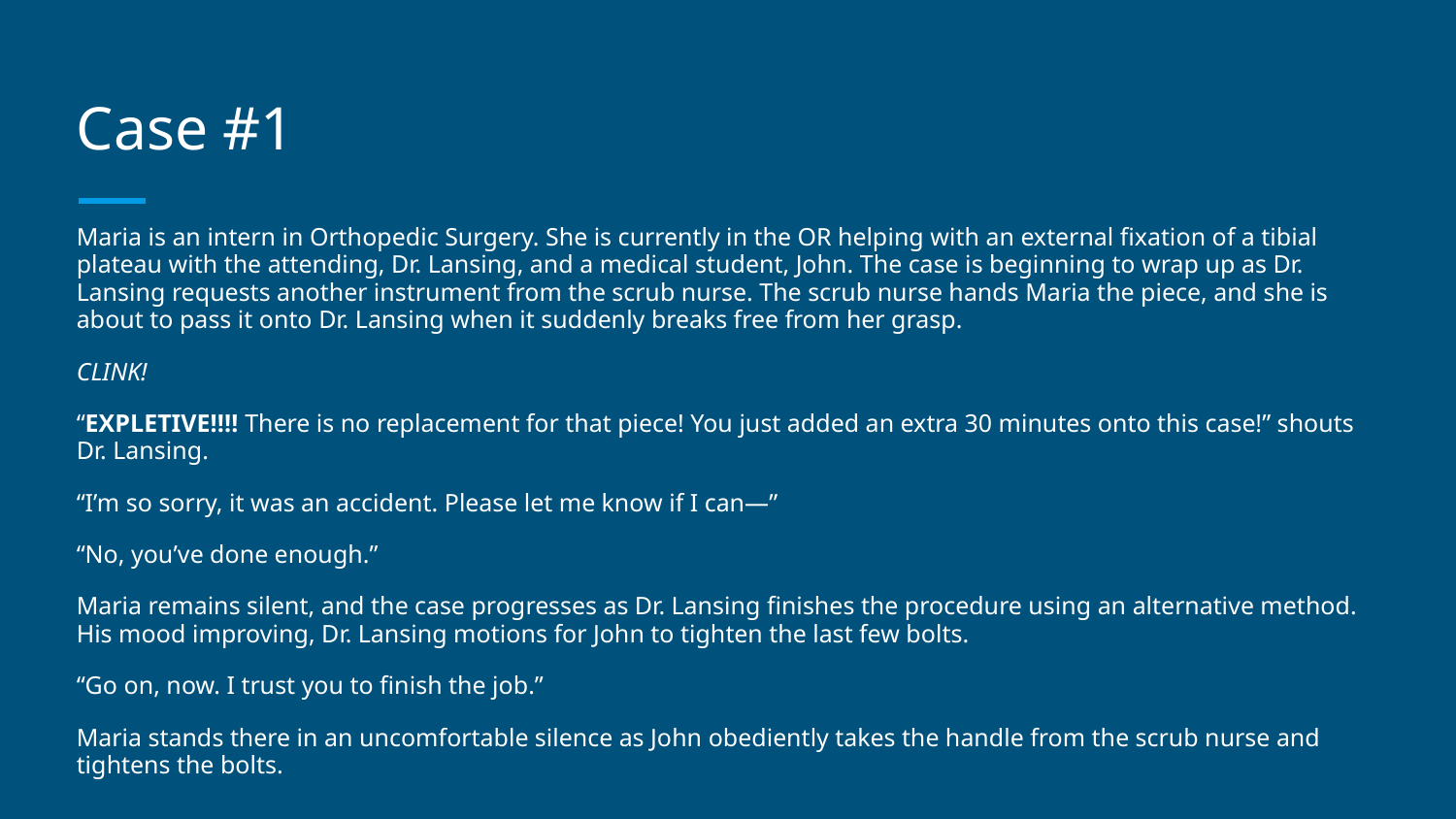

# Case #1
Maria is an intern in Orthopedic Surgery. She is currently in the OR helping with an external fixation of a tibial plateau with the attending, Dr. Lansing, and a medical student, John. The case is beginning to wrap up as Dr. Lansing requests another instrument from the scrub nurse. The scrub nurse hands Maria the piece, and she is about to pass it onto Dr. Lansing when it suddenly breaks free from her grasp.
CLINK!
“EXPLETIVE!!!! There is no replacement for that piece! You just added an extra 30 minutes onto this case!” shouts Dr. Lansing.
“I’m so sorry, it was an accident. Please let me know if I can—”
“No, you’ve done enough.”
Maria remains silent, and the case progresses as Dr. Lansing finishes the procedure using an alternative method. His mood improving, Dr. Lansing motions for John to tighten the last few bolts.
“Go on, now. I trust you to finish the job.”
Maria stands there in an uncomfortable silence as John obediently takes the handle from the scrub nurse and tightens the bolts.

## Slide 5
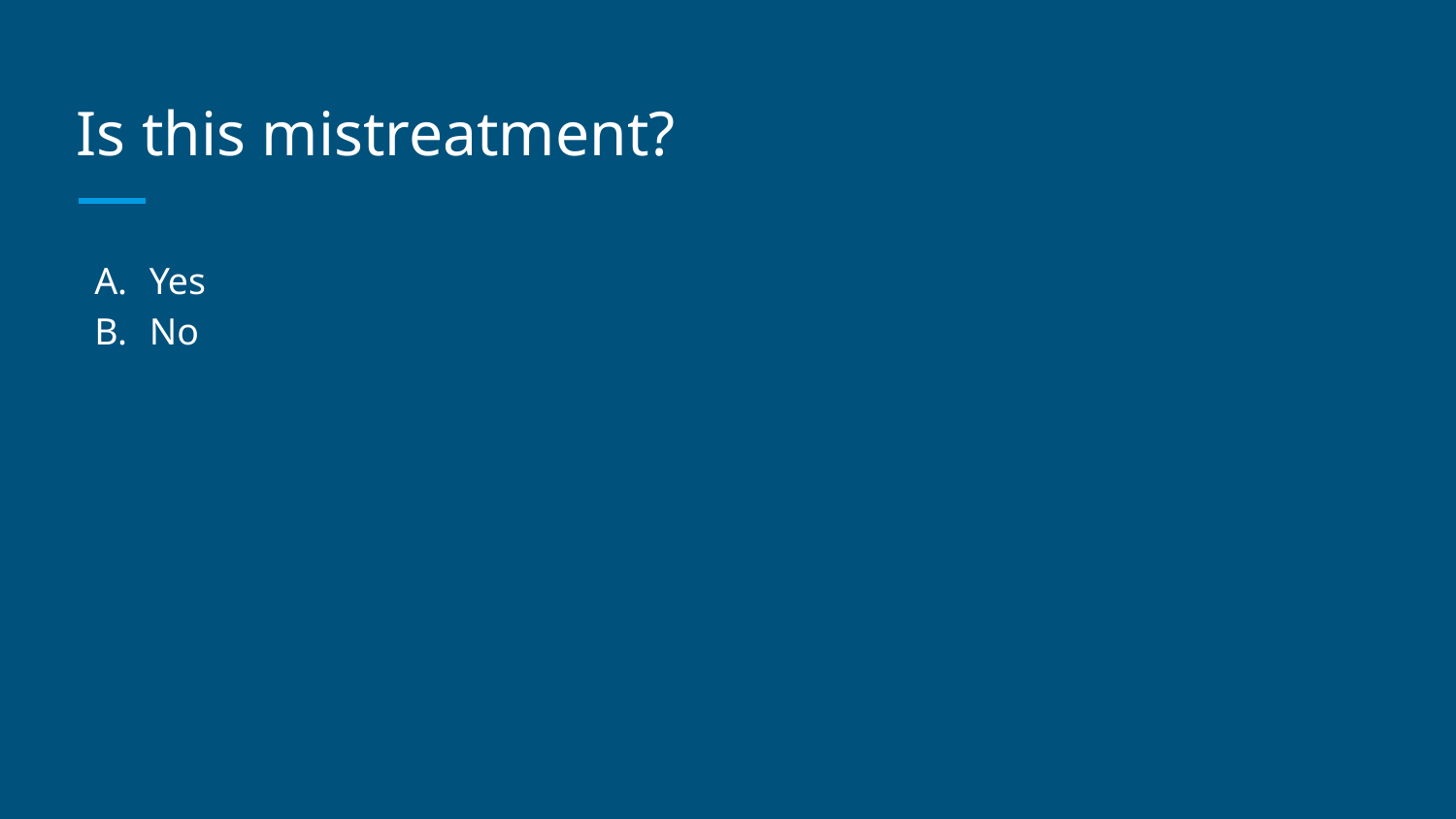

# Is this mistreatment?
Yes
No

## Slide 6
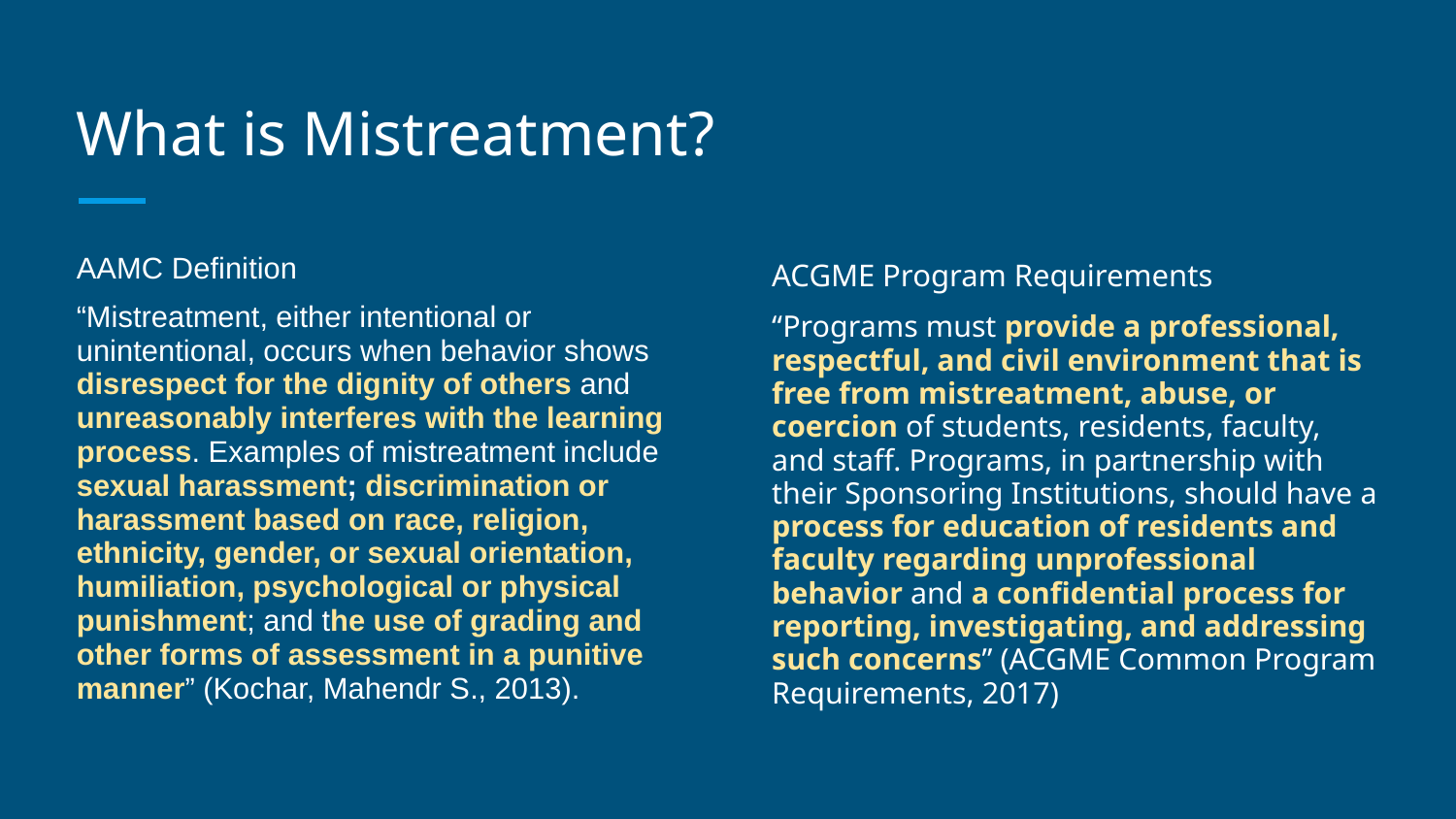

# What is Mistreatment?
AAMC Definition
“Mistreatment, either intentional or unintentional, occurs when behavior shows disrespect for the dignity of others and unreasonably interferes with the learning process. Examples of mistreatment include sexual harassment; discrimination or harassment based on race, religion, ethnicity, gender, or sexual orientation, humiliation, psychological or physical punishment; and the use of grading and other forms of assessment in a punitive manner” (Kochar, Mahendr S., 2013).
ACGME Program Requirements
“Programs must provide a professional, respectful, and civil environment that is free from mistreatment, abuse, or coercion of students, residents, faculty, and staff. Programs, in partnership with their Sponsoring Institutions, should have a process for education of residents and faculty regarding unprofessional behavior and a confidential process for reporting, investigating, and addressing such concerns” (ACGME Common Program Requirements, 2017)

## Slide 7
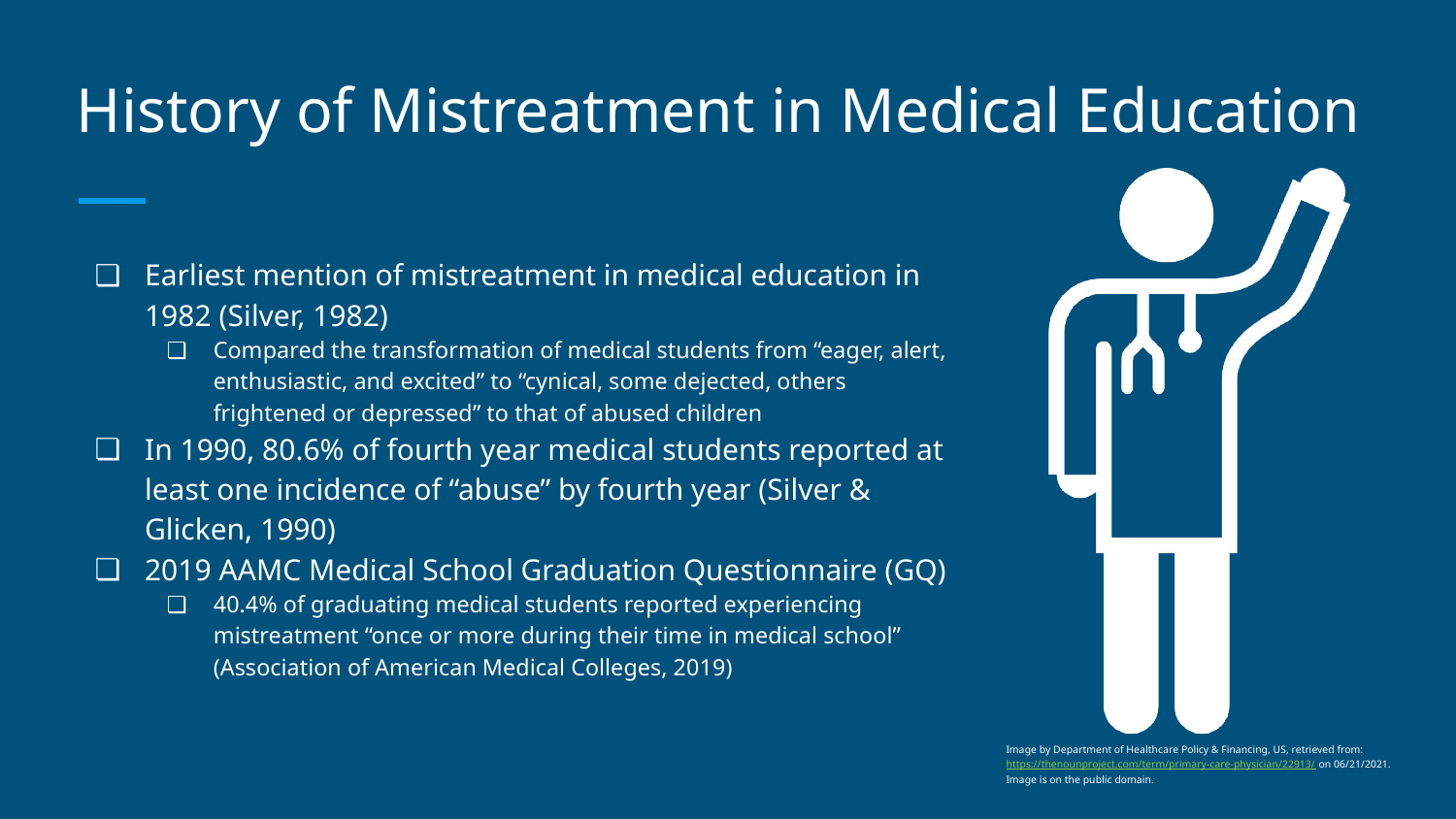

# History of Mistreatment in Medical Education
Earliest mention of mistreatment in medical education in 1982 (Silver, 1982)
Compared the transformation of medical students from “eager, alert, enthusiastic, and excited” to “cynical, some dejected, others frightened or depressed” to that of abused children
In 1990, 80.6% of fourth year medical students reported at least one incidence of “abuse” by fourth year (Silver & Glicken, 1990)
2019 AAMC Medical School Graduation Questionnaire (GQ)
40.4% of graduating medical students reported experiencing mistreatment “once or more during their time in medical school” (Association of American Medical Colleges, 2019)
Image by Department of Healthcare Policy & Financing, US, retrieved from: https://thenounproject.com/term/primary-care-physician/22913/ on 06/21/2021. Image is on the public domain.

## Slide 8
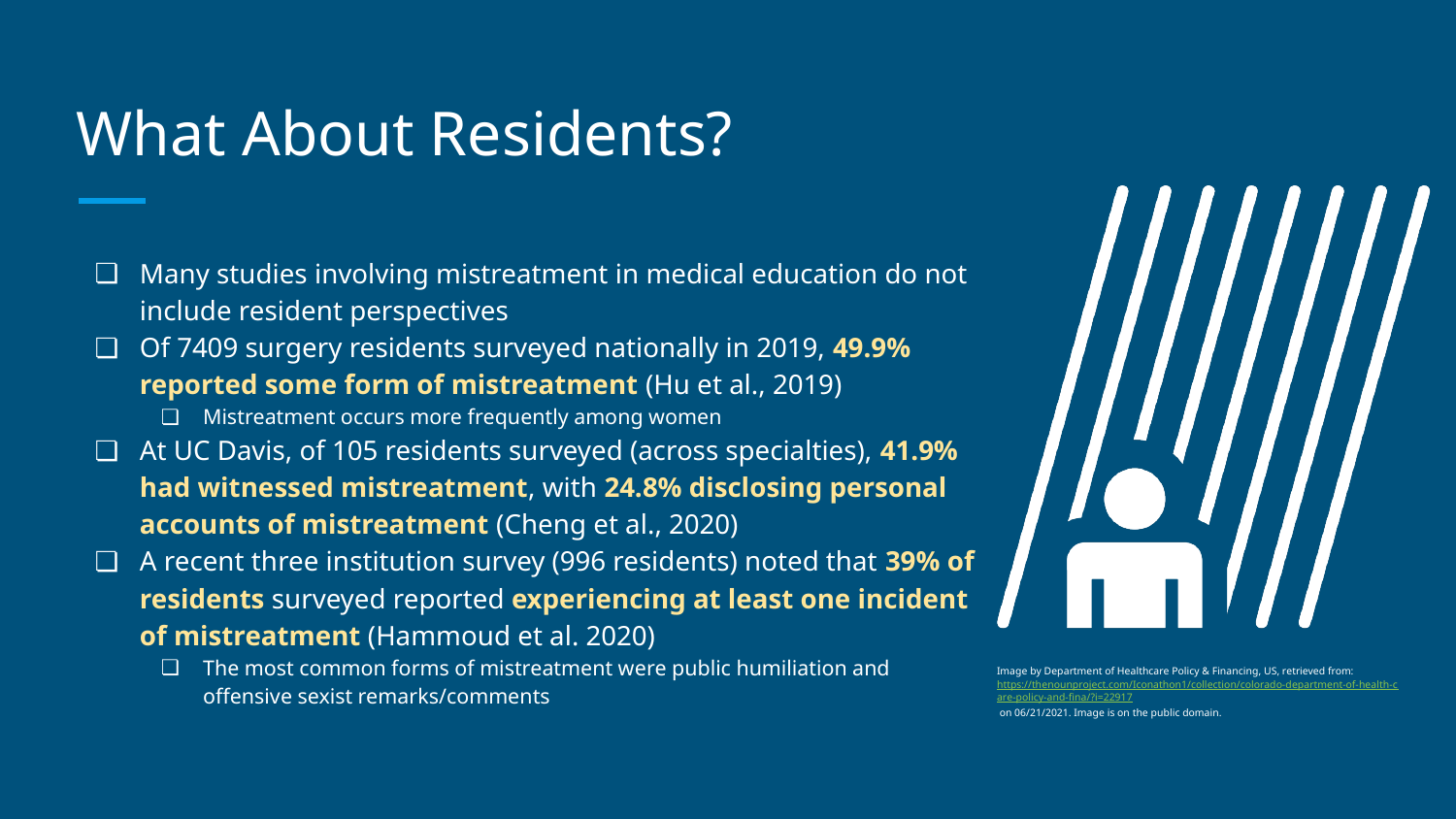

# What About Residents?
Many studies involving mistreatment in medical education do not include resident perspectives
Of 7409 surgery residents surveyed nationally in 2019, 49.9% reported some form of mistreatment (Hu et al., 2019)
Mistreatment occurs more frequently among women
At UC Davis, of 105 residents surveyed (across specialties), 41.9% had witnessed mistreatment, with 24.8% disclosing personal accounts of mistreatment (Cheng et al., 2020)
A recent three institution survey (996 residents) noted that 39% of residents surveyed reported experiencing at least one incident of mistreatment (Hammoud et al. 2020)
The most common forms of mistreatment were public humiliation and offensive sexist remarks/comments
Image by Department of Healthcare Policy & Financing, US, retrieved from: https://thenounproject.com/Iconathon1/collection/colorado-department-of-health-care-policy-and-fina/?i=22917 on 06/21/2021. Image is on the public domain.

## Slide 9
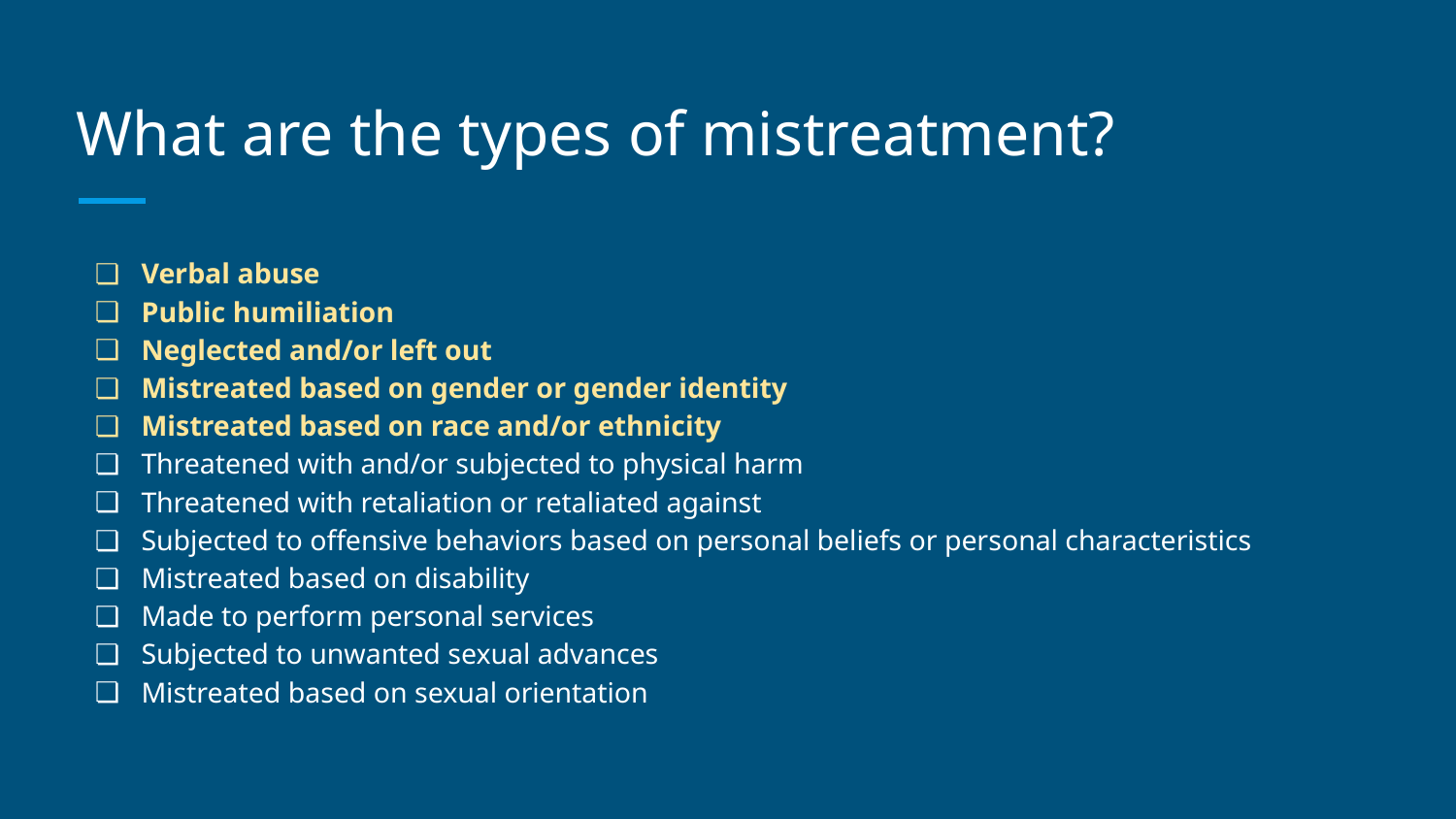

# What are the types of mistreatment?
Verbal abuse
Public humiliation
Neglected and/or left out
Mistreated based on gender or gender identity
Mistreated based on race and/or ethnicity
Threatened with and/or subjected to physical harm
Threatened with retaliation or retaliated against
Subjected to offensive behaviors based on personal beliefs or personal characteristics
Mistreated based on disability
Made to perform personal services
Subjected to unwanted sexual advances
Mistreated based on sexual orientation

## Slide 10
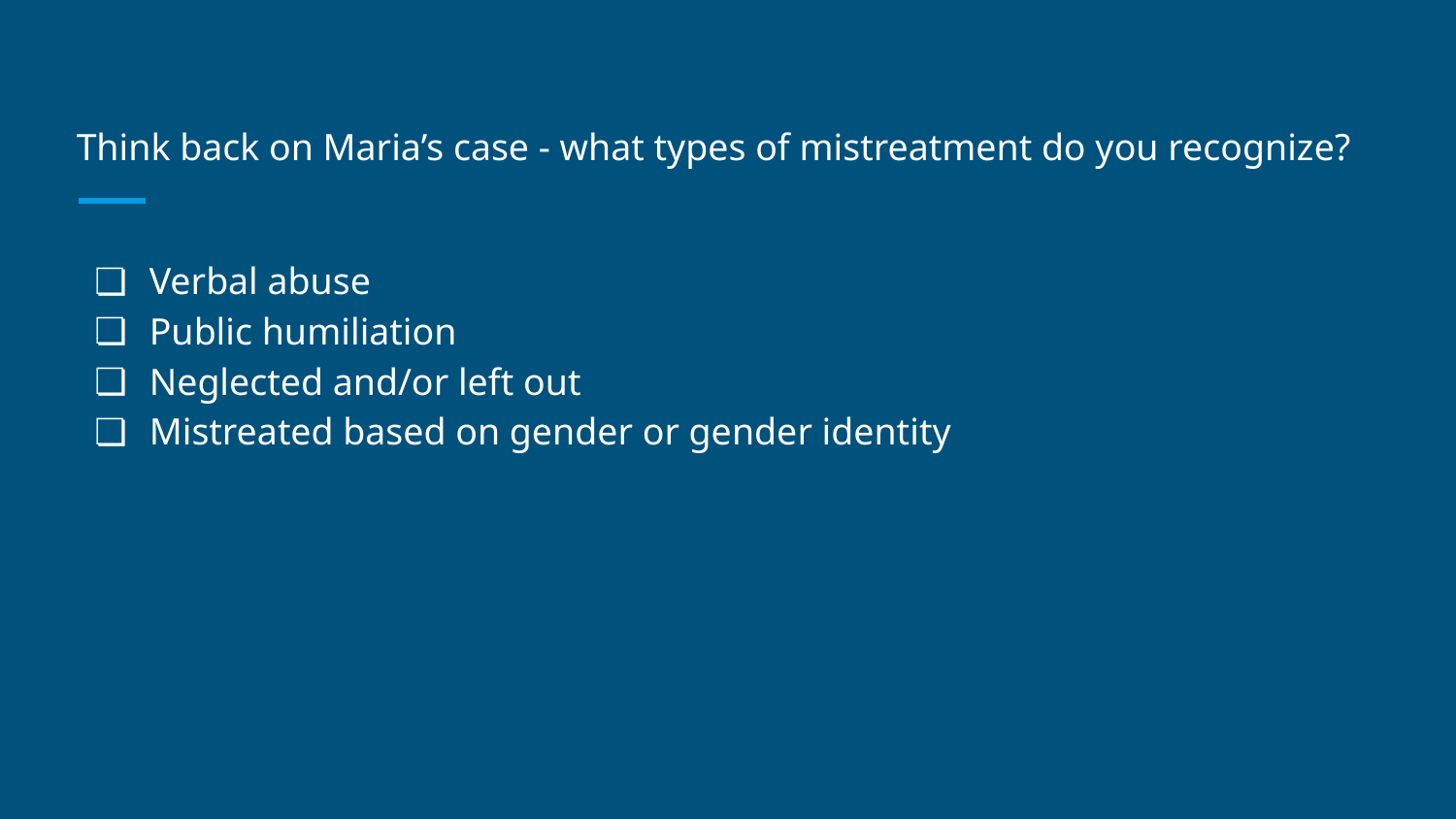

# Think back on Maria’s case - what types of mistreatment do you recognize?
Verbal abuse
Public humiliation
Neglected and/or left out
Mistreated based on gender or gender identity

## Slide 11
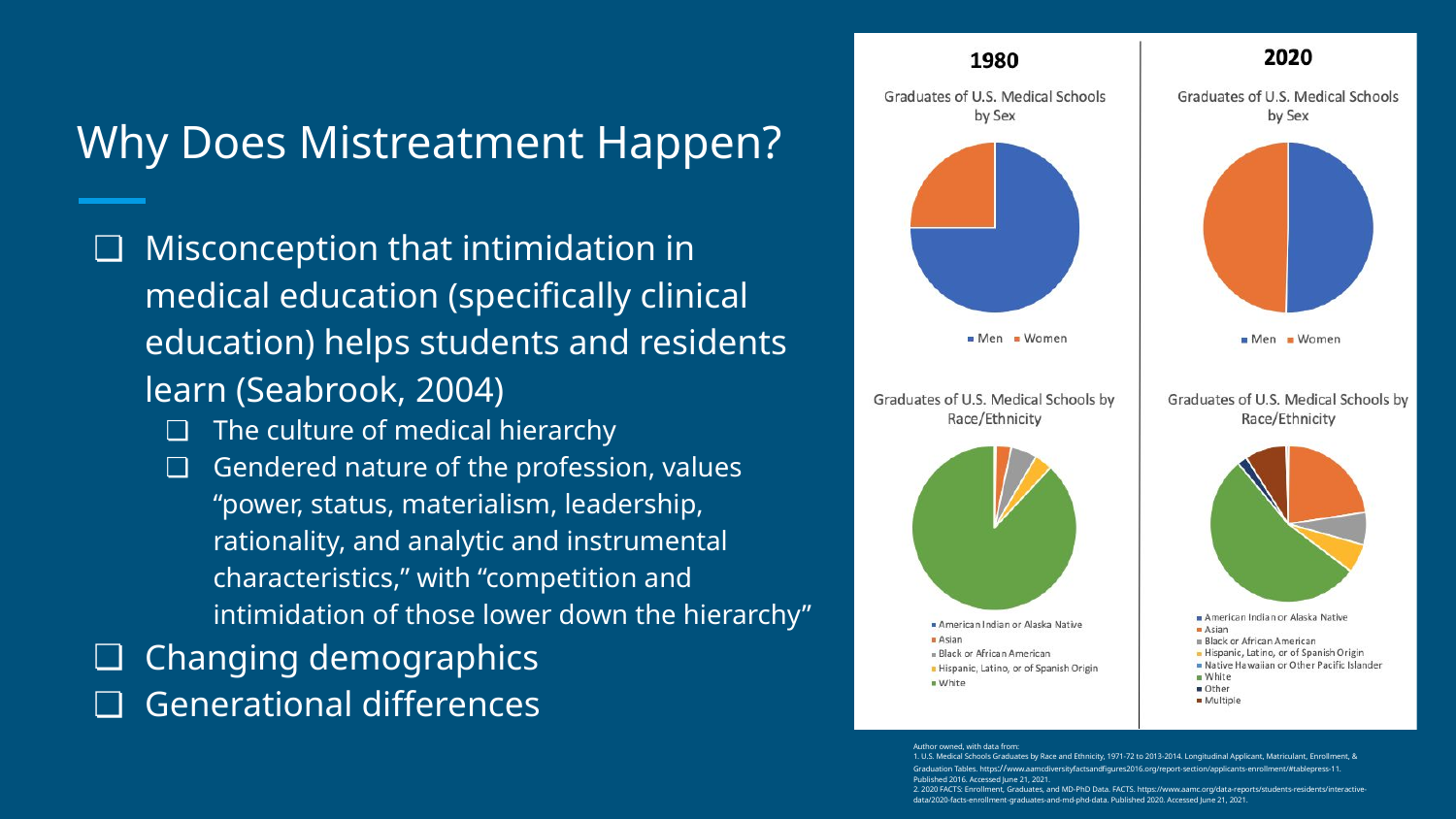

# Why Does Mistreatment Happen?
Misconception that intimidation in medical education (specifically clinical education) helps students and residents learn (Seabrook, 2004)
The culture of medical hierarchy
Gendered nature of the profession, values “power, status, materialism, leadership, rationality, and analytic and instrumental characteristics,” with “competition and intimidation of those lower down the hierarchy”
Changing demographics
Generational differences
Author owned, with data from:
1. U.S. Medical Schools Graduates by Race and Ethnicity, 1971-72 to 2013-2014. Longitudinal Applicant, Matriculant, Enrollment, & Graduation Tables. https://www.aamcdiversityfactsandfigures2016.org/report-section/applicants-enrollment/#tablepress-11. Published 2016. Accessed June 21, 2021.
2. 2020 FACTS: Enrollment, Graduates, and MD-PhD Data. FACTS. https://www.aamc.org/data-reports/students-residents/interactive-data/2020-facts-enrollment-graduates-and-md-phd-data. Published 2020. Accessed June 21, 2021.

## Slide 12
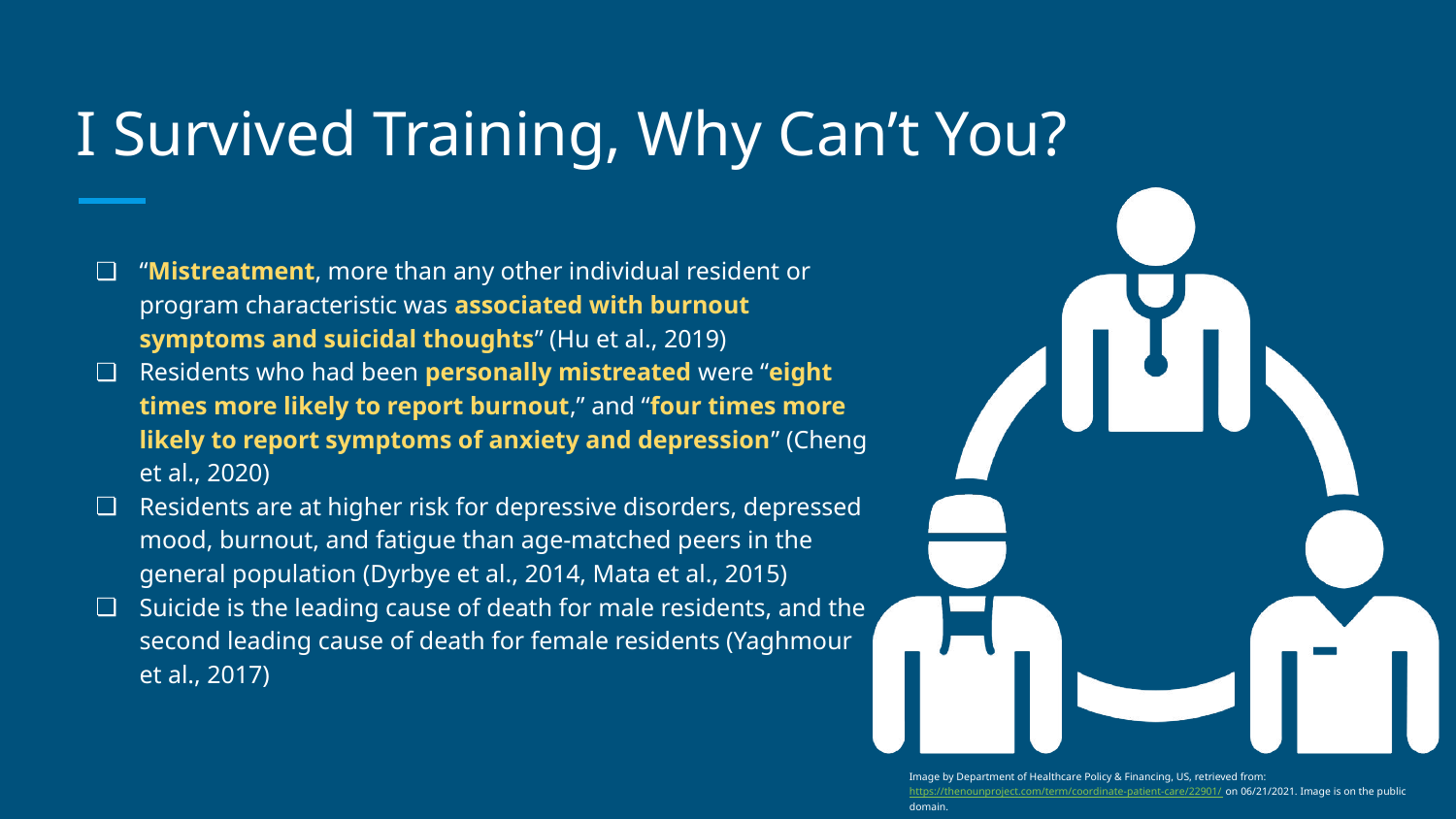

# I Survived Training, Why Can’t You?
“Mistreatment, more than any other individual resident or program characteristic was associated with burnout symptoms and suicidal thoughts” (Hu et al., 2019)
Residents who had been personally mistreated were “eight times more likely to report burnout,” and “four times more likely to report symptoms of anxiety and depression” (Cheng et al., 2020)
Residents are at higher risk for depressive disorders, depressed mood, burnout, and fatigue than age-matched peers in the general population (Dyrbye et al., 2014, Mata et al., 2015)
Suicide is the leading cause of death for male residents, and the second leading cause of death for female residents (Yaghmour et al., 2017)
Image by Department of Healthcare Policy & Financing, US, retrieved from: https://thenounproject.com/term/coordinate-patient-care/22901/ on 06/21/2021. Image is on the public domain.

## Slide 13
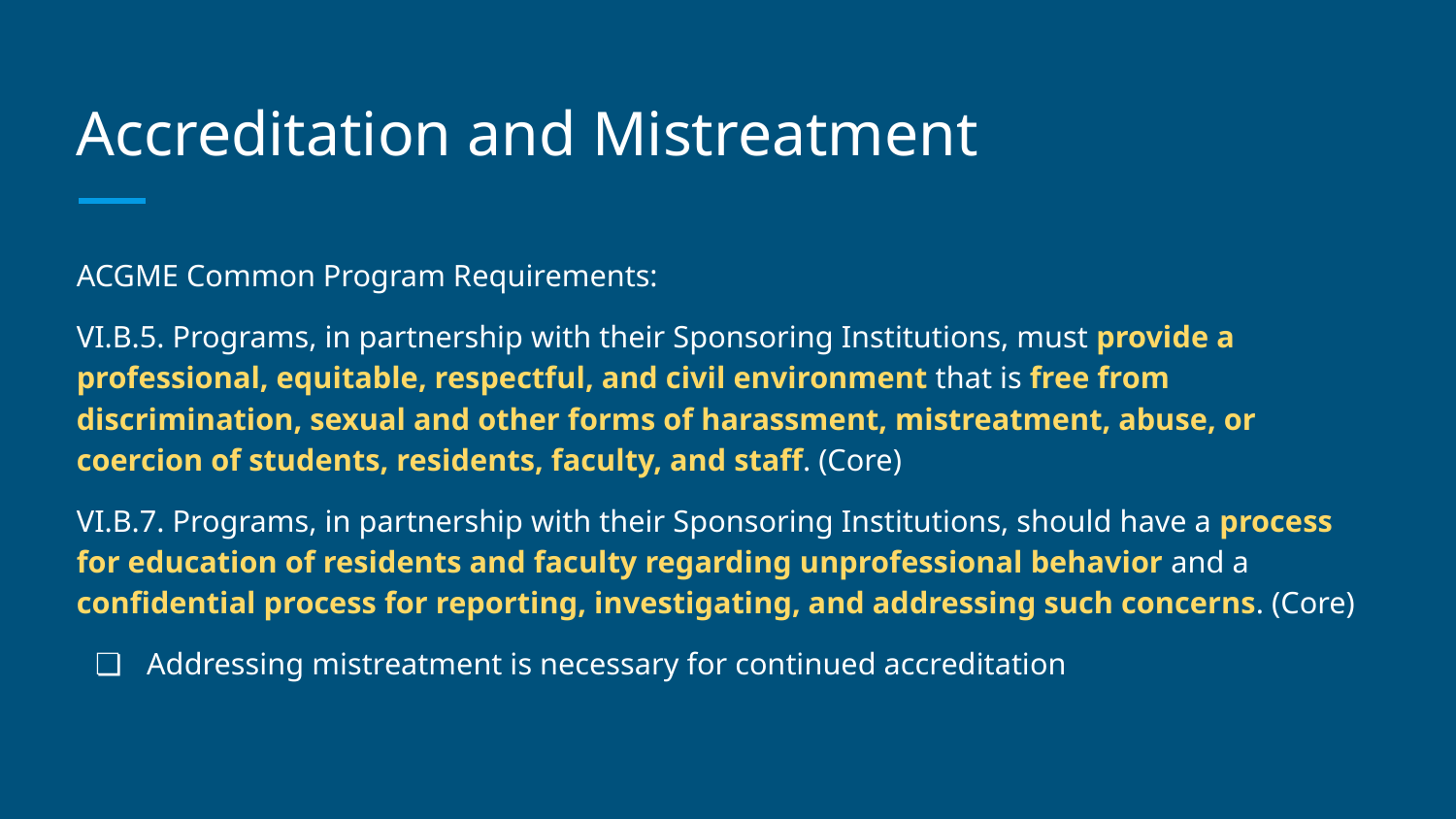

# Accreditation and Mistreatment
ACGME Common Program Requirements:
VI.B.5. Programs, in partnership with their Sponsoring Institutions, must provide a professional, equitable, respectful, and civil environment that is free from discrimination, sexual and other forms of harassment, mistreatment, abuse, or coercion of students, residents, faculty, and staff. (Core)
VI.B.7. Programs, in partnership with their Sponsoring Institutions, should have a process for education of residents and faculty regarding unprofessional behavior and a confidential process for reporting, investigating, and addressing such concerns. (Core)
Addressing mistreatment is necessary for continued accreditation

## Slide 14
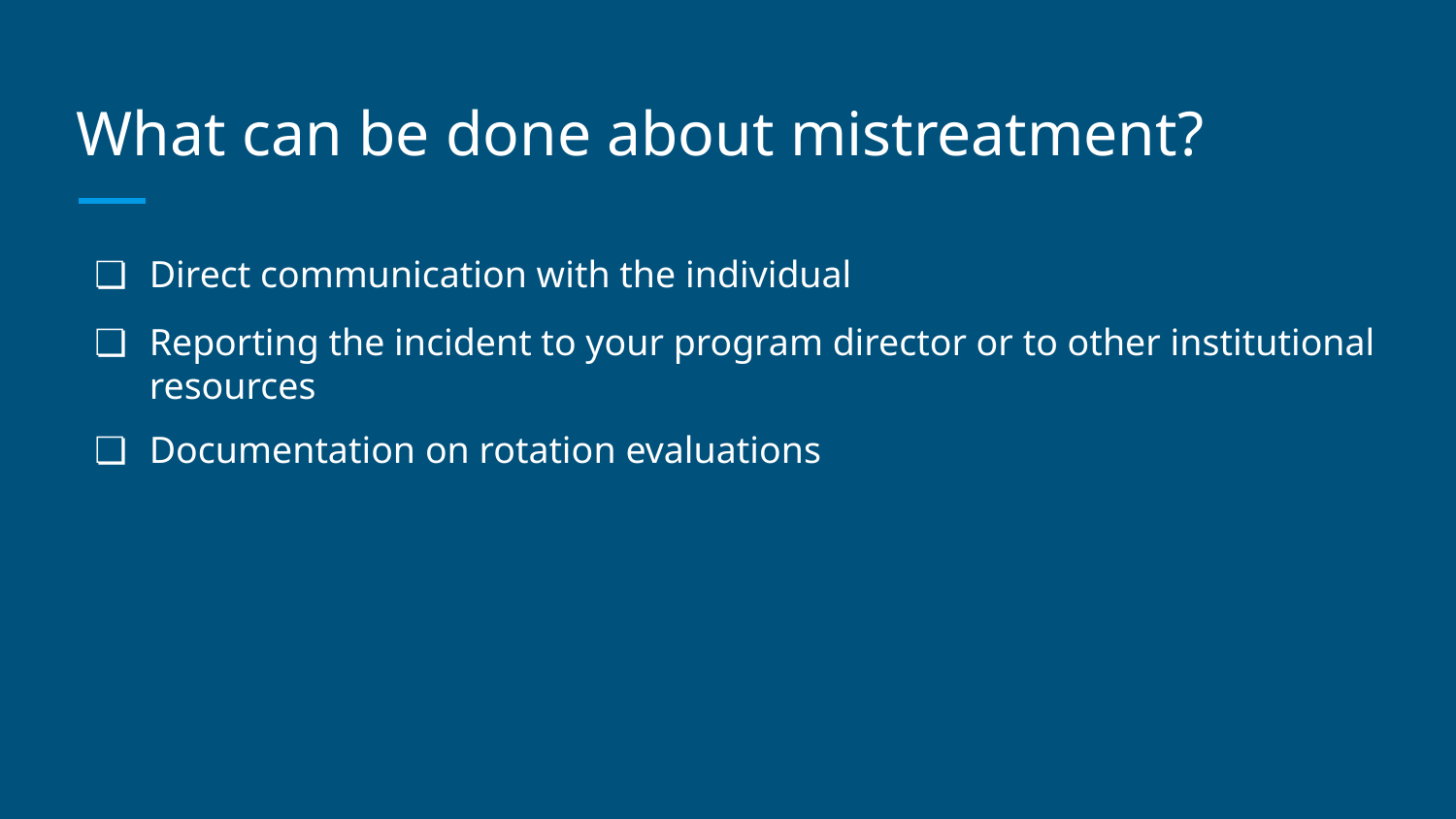

# What can be done about mistreatment?
Direct communication with the individual
Reporting the incident to your program director or to other institutional resources
Documentation on rotation evaluations

## Slide 15
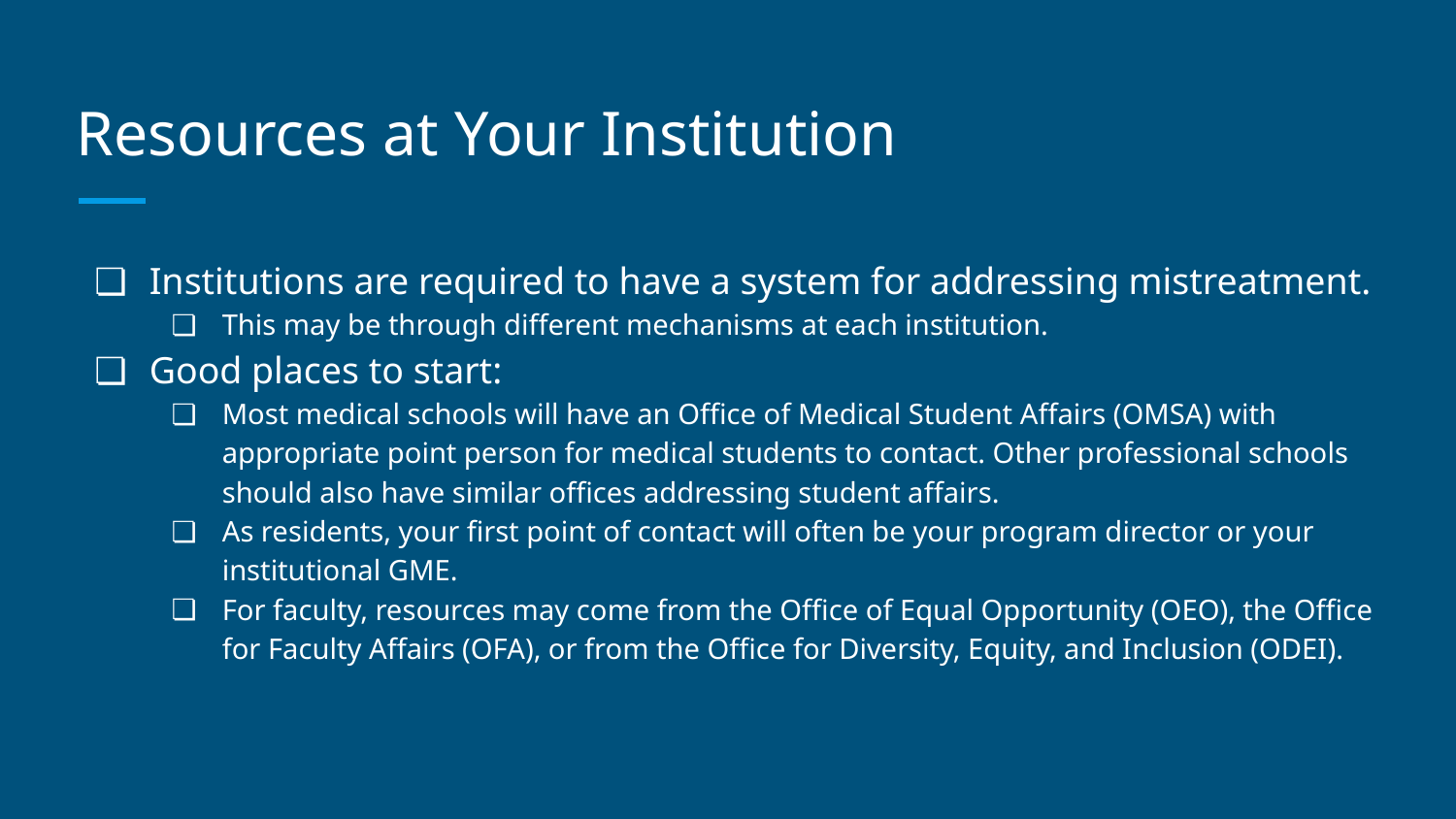

# Resources at Your Institution
Institutions are required to have a system for addressing mistreatment.
This may be through different mechanisms at each institution.
Good places to start:
Most medical schools will have an Office of Medical Student Affairs (OMSA) with appropriate point person for medical students to contact. Other professional schools should also have similar offices addressing student affairs.
As residents, your first point of contact will often be your program director or your institutional GME.
For faculty, resources may come from the Office of Equal Opportunity (OEO), the Office for Faculty Affairs (OFA), or from the Office for Diversity, Equity, and Inclusion (ODEI).

## Slide 16
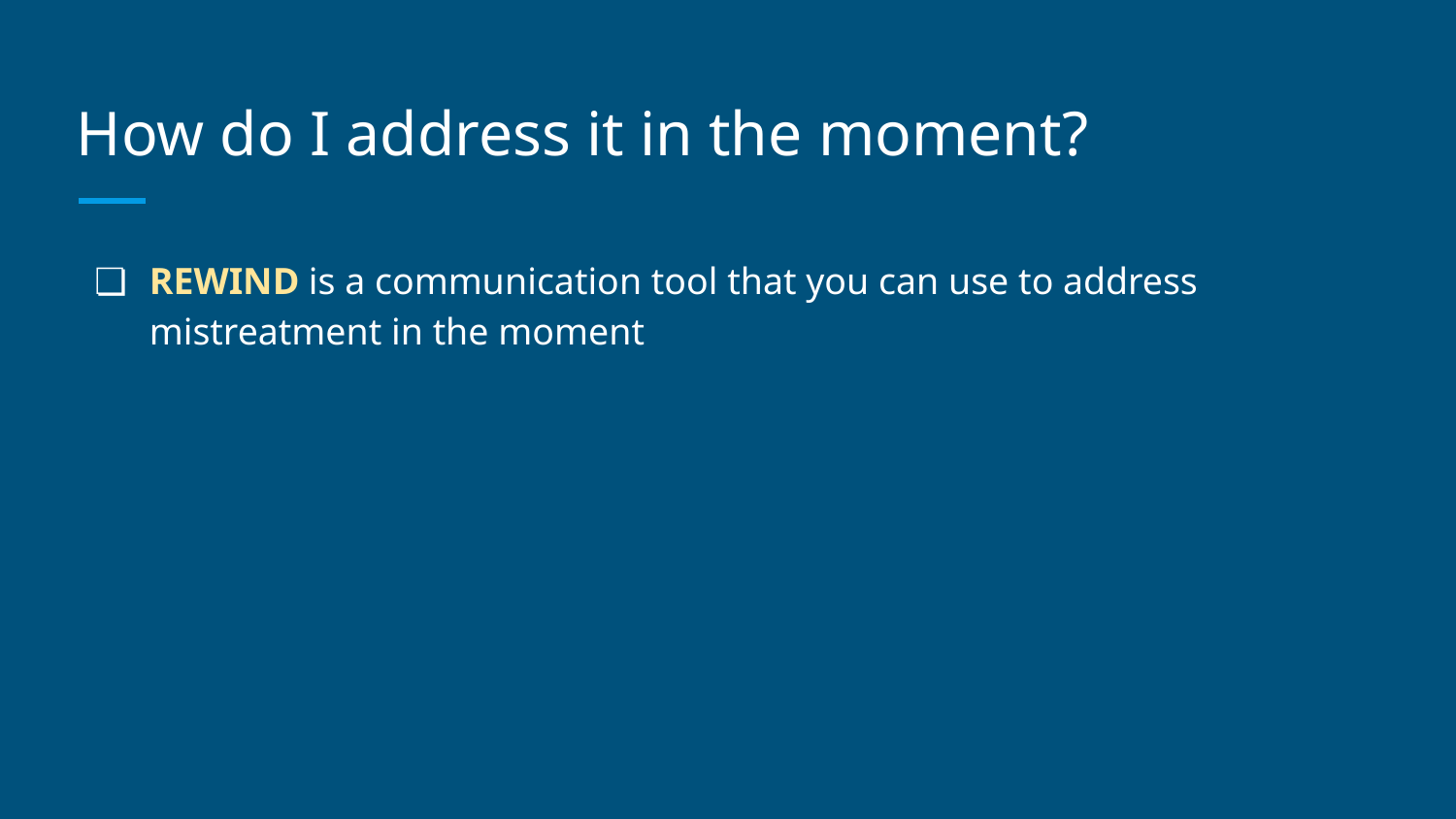

# How do I address it in the moment?
REWIND is a communication tool that you can use to address mistreatment in the moment

## Slide 17
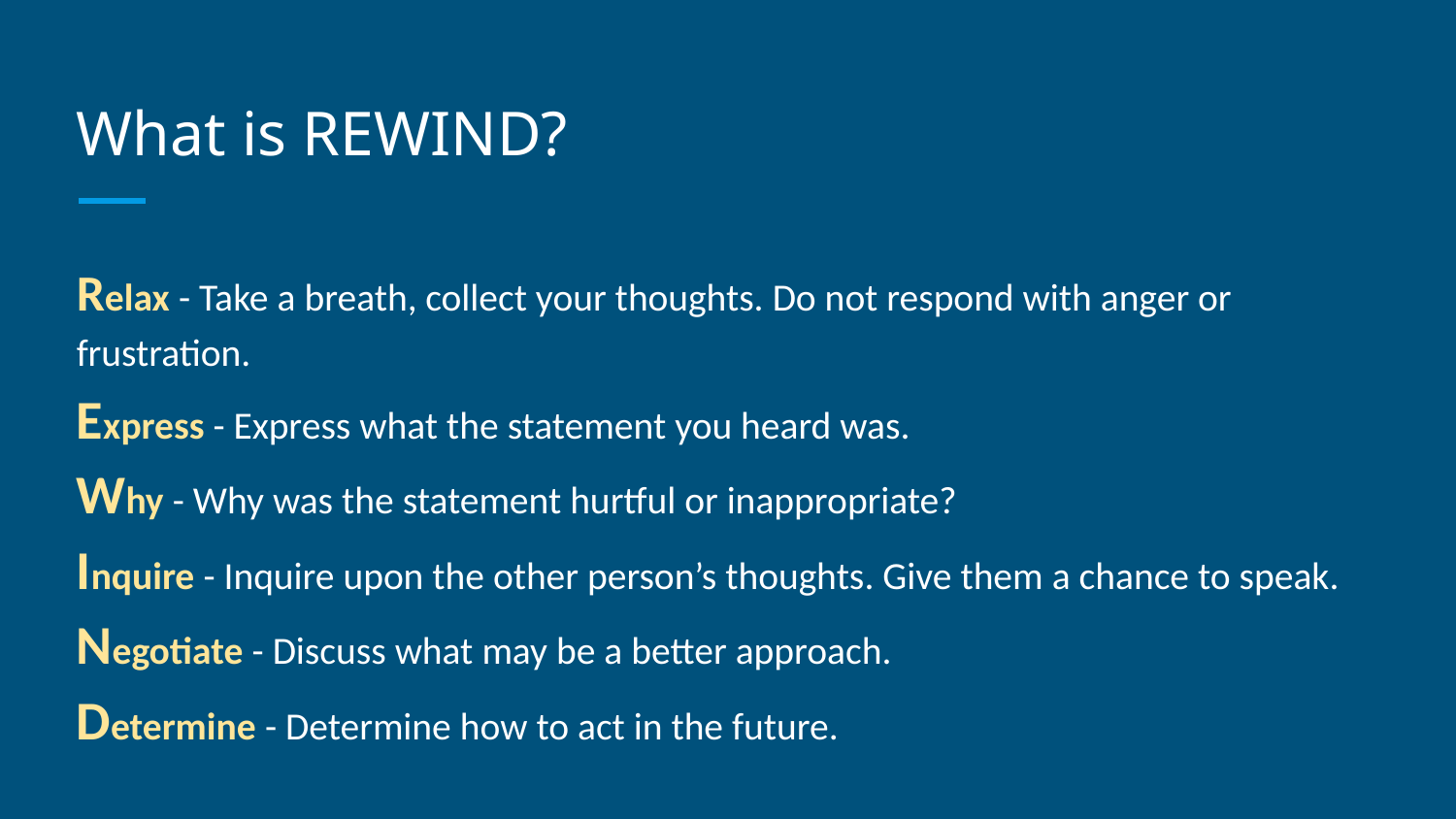

# What is REWIND?
Relax - Take a breath, collect your thoughts. Do not respond with anger or frustration.
Express - Express what the statement you heard was.
Why - Why was the statement hurtful or inappropriate?
Inquire - Inquire upon the other person’s thoughts. Give them a chance to speak.
Negotiate - Discuss what may be a better approach.
Determine - Determine how to act in the future.

## Slide 18
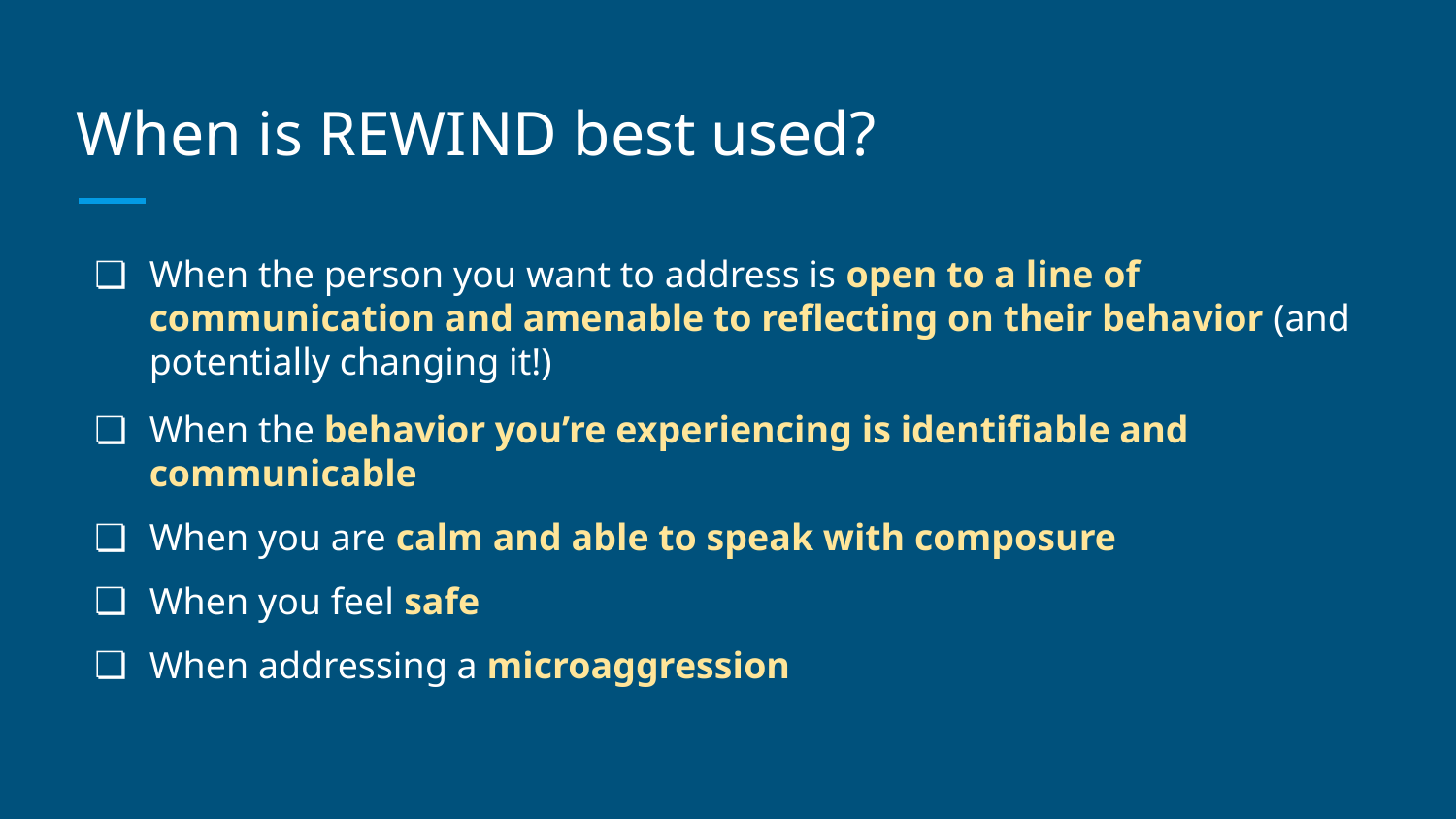

# When is REWIND best used?
When the person you want to address is open to a line of communication and amenable to reflecting on their behavior (and potentially changing it!)
When the behavior you’re experiencing is identifiable and communicable
When you are calm and able to speak with composure
When you feel safe
When addressing a microaggression

## Slide 19
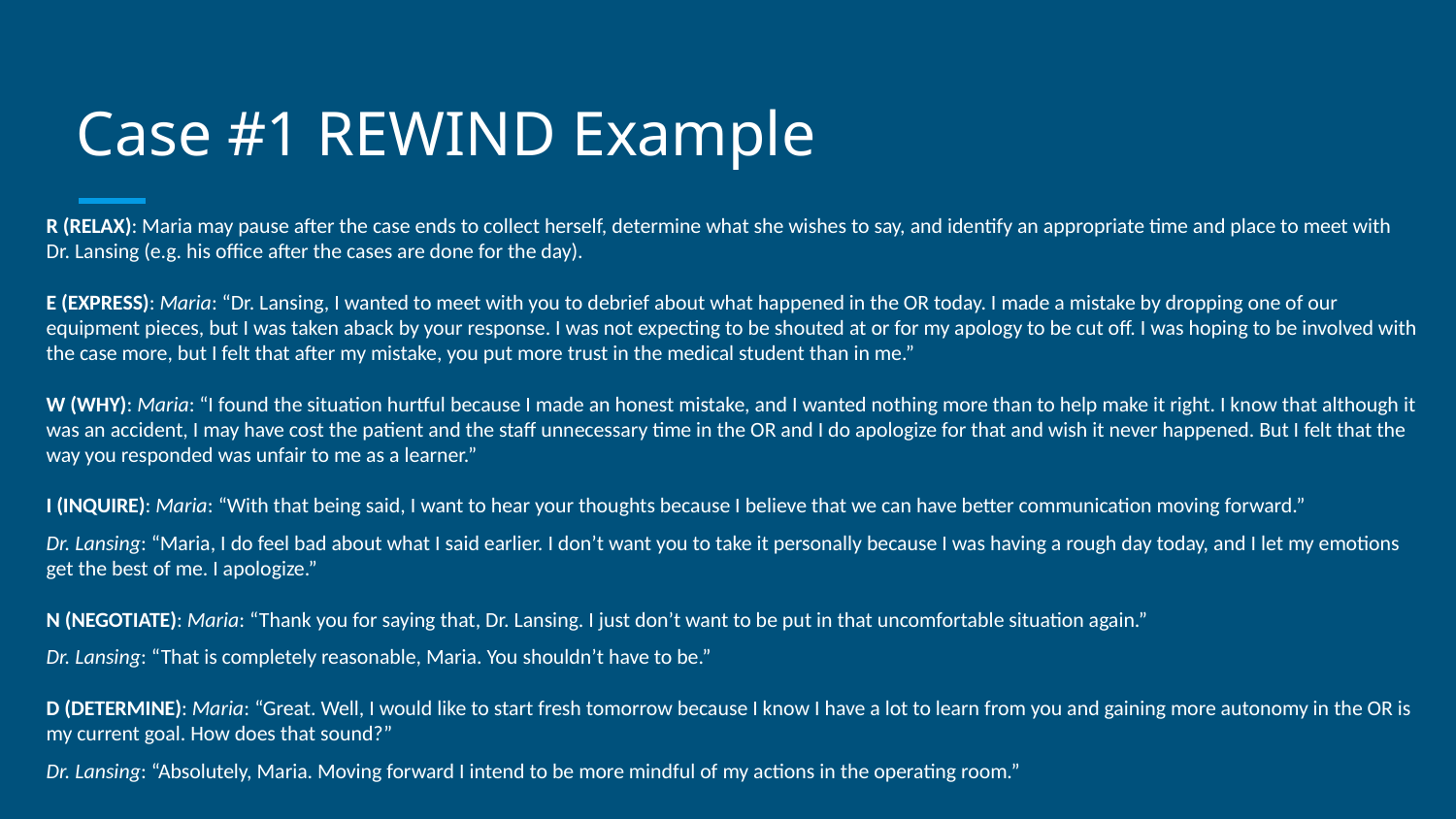

# Case #1 REWIND Example
R (RELAX): Maria may pause after the case ends to collect herself, determine what she wishes to say, and identify an appropriate time and place to meet with Dr. Lansing (e.g. his office after the cases are done for the day).
E (EXPRESS): Maria: “Dr. Lansing, I wanted to meet with you to debrief about what happened in the OR today. I made a mistake by dropping one of our equipment pieces, but I was taken aback by your response. I was not expecting to be shouted at or for my apology to be cut off. I was hoping to be involved with the case more, but I felt that after my mistake, you put more trust in the medical student than in me.”
W (WHY): Maria: “I found the situation hurtful because I made an honest mistake, and I wanted nothing more than to help make it right. I know that although it was an accident, I may have cost the patient and the staff unnecessary time in the OR and I do apologize for that and wish it never happened. But I felt that the way you responded was unfair to me as a learner.”
I (INQUIRE): Maria: “With that being said, I want to hear your thoughts because I believe that we can have better communication moving forward.”
Dr. Lansing: “Maria, I do feel bad about what I said earlier. I don’t want you to take it personally because I was having a rough day today, and I let my emotions get the best of me. I apologize.”
N (NEGOTIATE): Maria: “Thank you for saying that, Dr. Lansing. I just don’t want to be put in that uncomfortable situation again.”
Dr. Lansing: “That is completely reasonable, Maria. You shouldn’t have to be.”
D (DETERMINE): Maria: “Great. Well, I would like to start fresh tomorrow because I know I have a lot to learn from you and gaining more autonomy in the OR is my current goal. How does that sound?”
Dr. Lansing: “Absolutely, Maria. Moving forward I intend to be more mindful of my actions in the operating room.”

## Slide 20
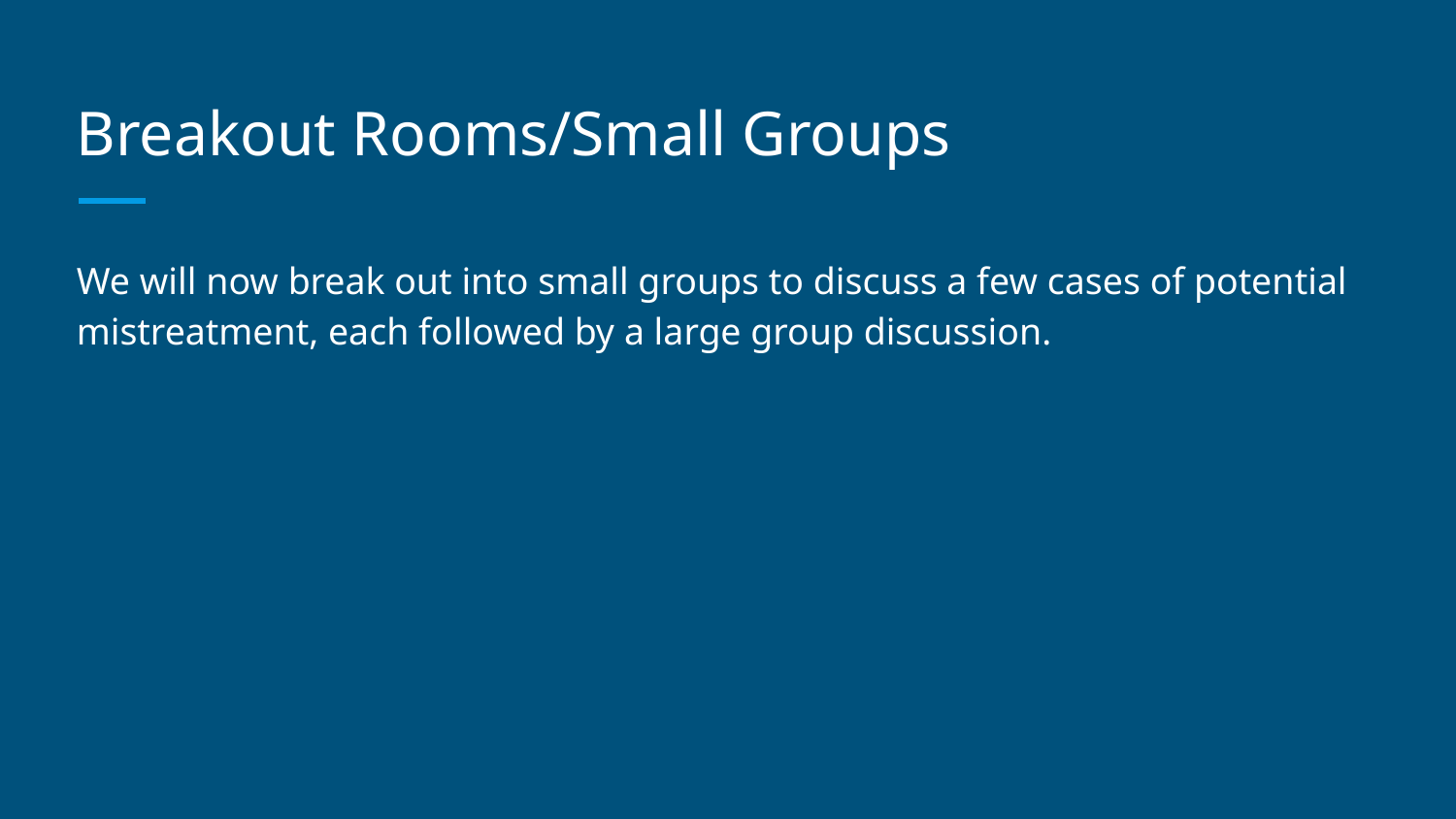

# Breakout Rooms/Small Groups
We will now break out into small groups to discuss a few cases of potential mistreatment, each followed by a large group discussion.

## Slide 21
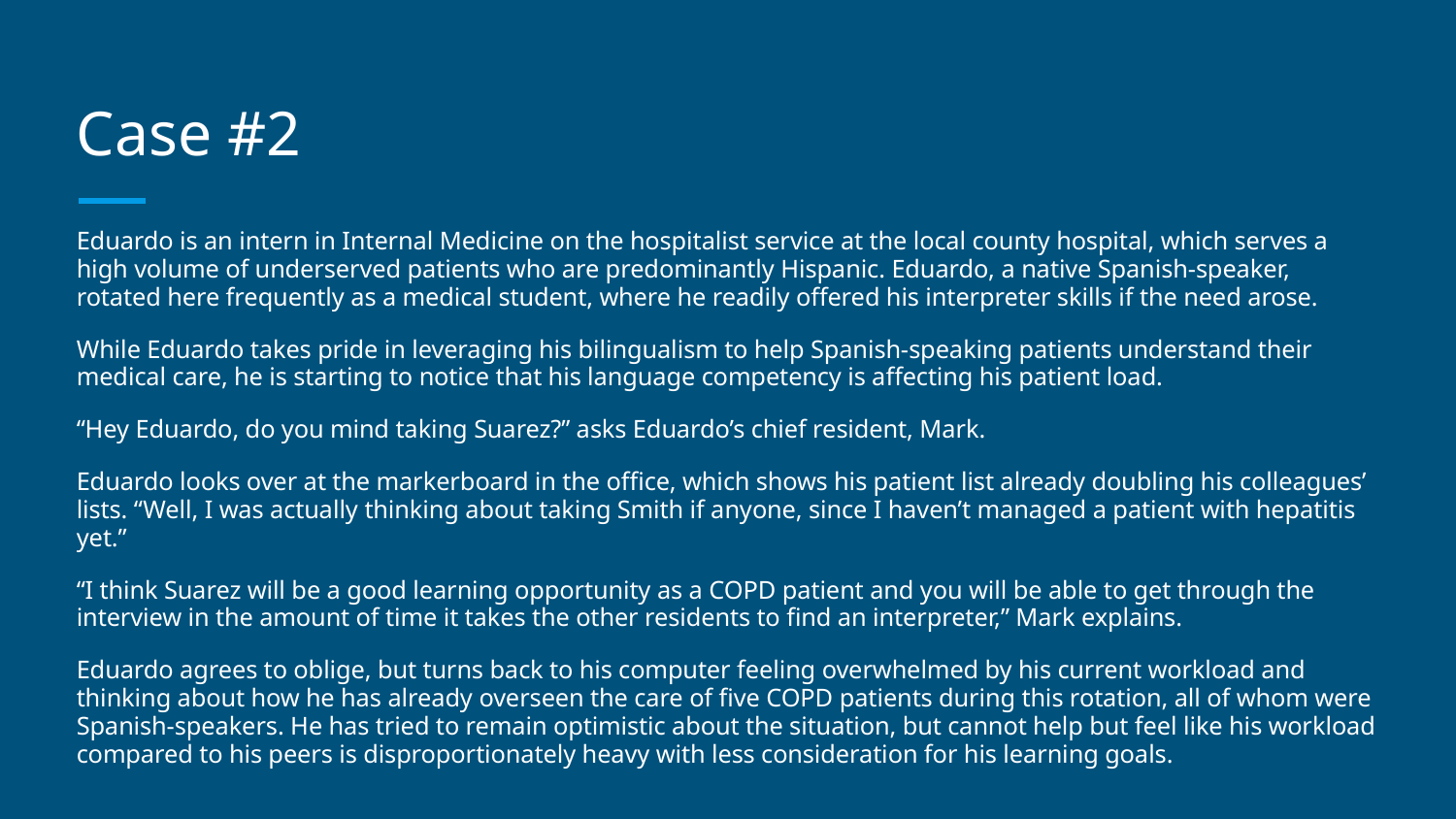

# Case #2
Eduardo is an intern in Internal Medicine on the hospitalist service at the local county hospital, which serves a high volume of underserved patients who are predominantly Hispanic. Eduardo, a native Spanish-speaker, rotated here frequently as a medical student, where he readily offered his interpreter skills if the need arose.
While Eduardo takes pride in leveraging his bilingualism to help Spanish-speaking patients understand their medical care, he is starting to notice that his language competency is affecting his patient load.
“Hey Eduardo, do you mind taking Suarez?” asks Eduardo’s chief resident, Mark.
Eduardo looks over at the markerboard in the office, which shows his patient list already doubling his colleagues’ lists. “Well, I was actually thinking about taking Smith if anyone, since I haven’t managed a patient with hepatitis yet.”
“I think Suarez will be a good learning opportunity as a COPD patient and you will be able to get through the interview in the amount of time it takes the other residents to find an interpreter,” Mark explains.
Eduardo agrees to oblige, but turns back to his computer feeling overwhelmed by his current workload and thinking about how he has already overseen the care of five COPD patients during this rotation, all of whom were Spanish-speakers. He has tried to remain optimistic about the situation, but cannot help but feel like his workload compared to his peers is disproportionately heavy with less consideration for his learning goals.

## Slide 22
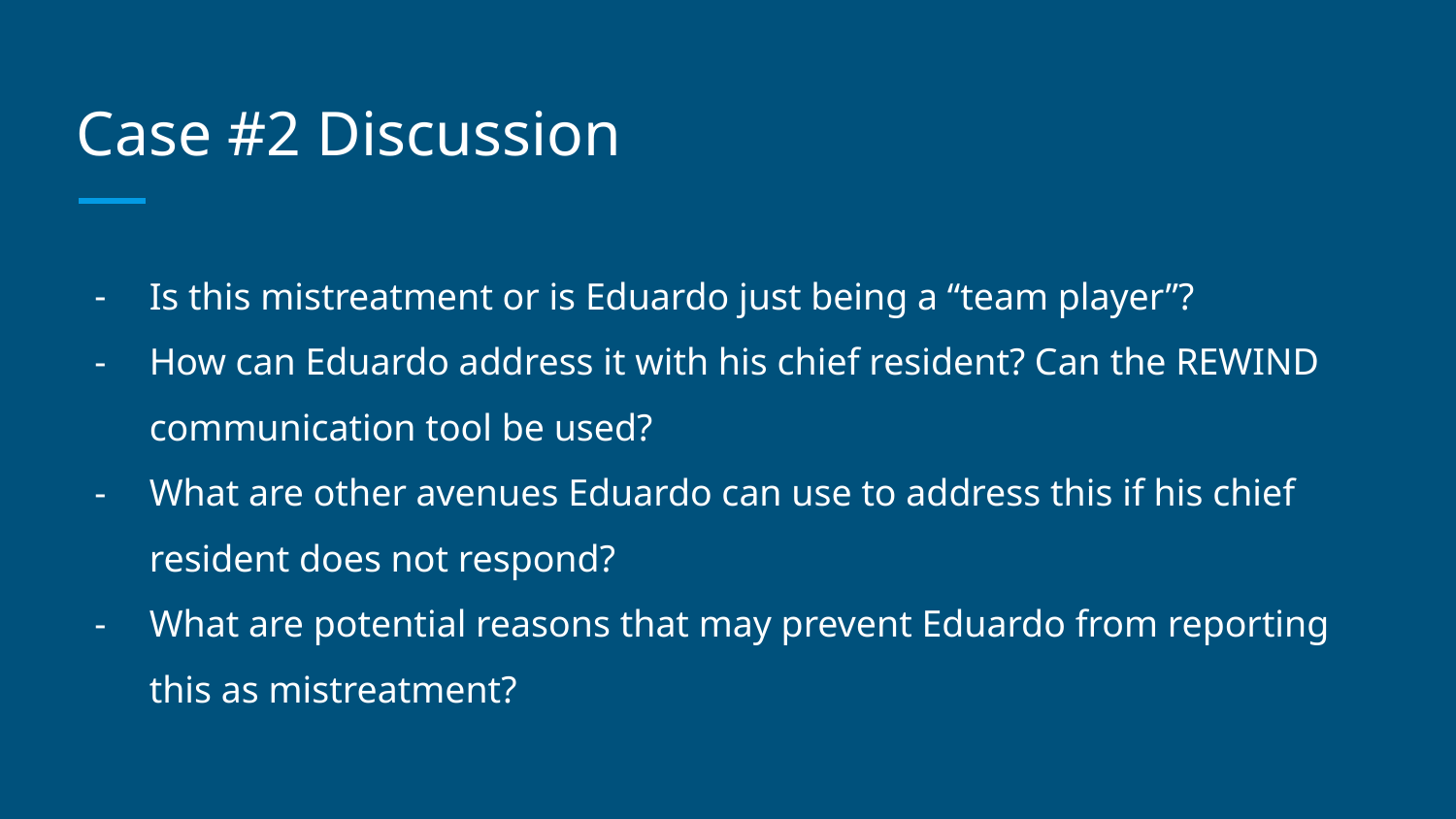

# Case #2 Discussion
Is this mistreatment or is Eduardo just being a “team player”?
How can Eduardo address it with his chief resident? Can the REWIND communication tool be used?
What are other avenues Eduardo can use to address this if his chief resident does not respond?
What are potential reasons that may prevent Eduardo from reporting this as mistreatment?

## Slide 23
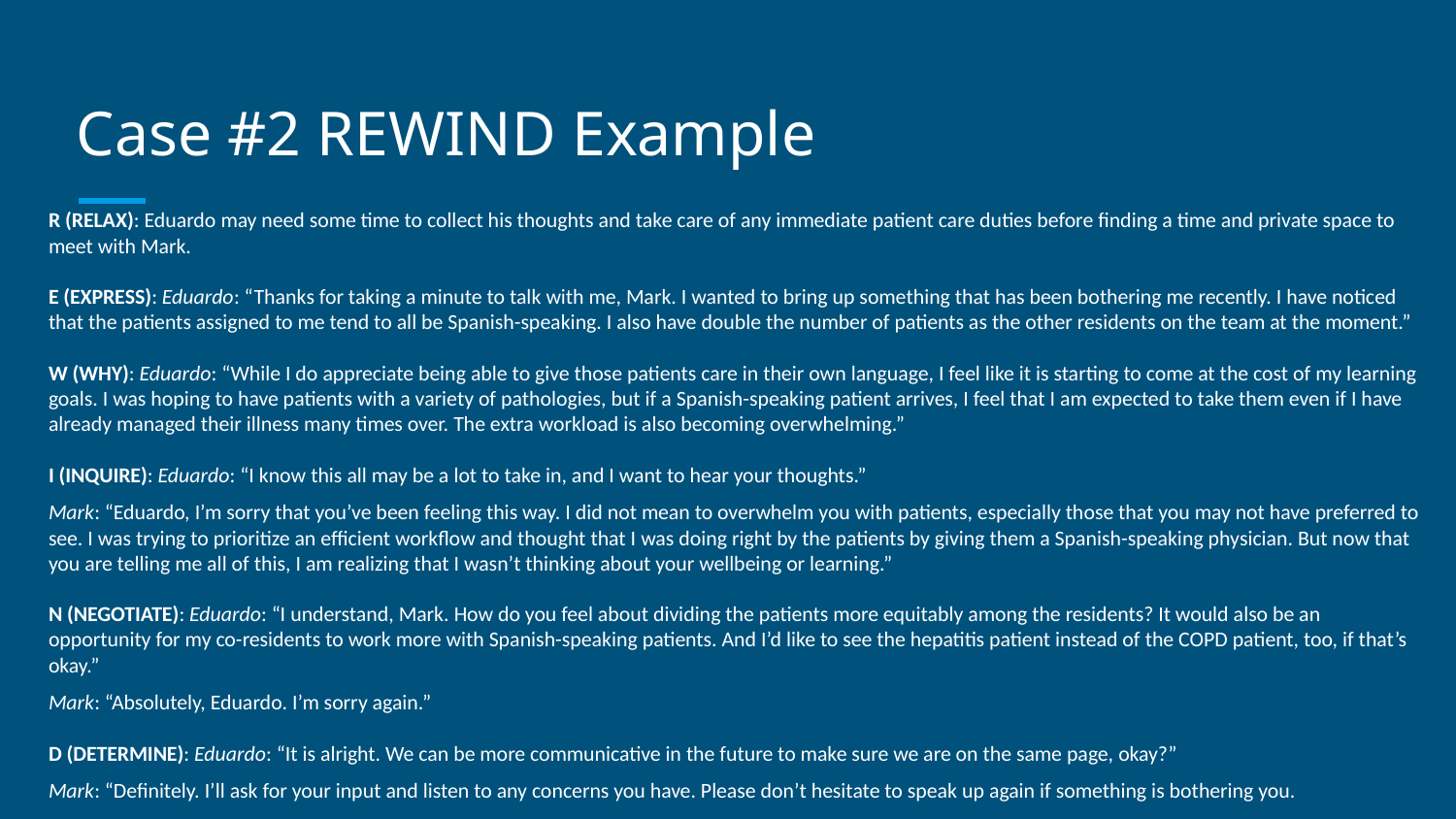

# Case #2 REWIND Example
R (RELAX): Eduardo may need some time to collect his thoughts and take care of any immediate patient care duties before finding a time and private space to meet with Mark.
E (EXPRESS): Eduardo: “Thanks for taking a minute to talk with me, Mark. I wanted to bring up something that has been bothering me recently. I have noticed that the patients assigned to me tend to all be Spanish-speaking. I also have double the number of patients as the other residents on the team at the moment.”
W (WHY): Eduardo: “While I do appreciate being able to give those patients care in their own language, I feel like it is starting to come at the cost of my learning goals. I was hoping to have patients with a variety of pathologies, but if a Spanish-speaking patient arrives, I feel that I am expected to take them even if I have already managed their illness many times over. The extra workload is also becoming overwhelming.”
I (INQUIRE): Eduardo: “I know this all may be a lot to take in, and I want to hear your thoughts.”
Mark: “Eduardo, I’m sorry that you’ve been feeling this way. I did not mean to overwhelm you with patients, especially those that you may not have preferred to see. I was trying to prioritize an efficient workflow and thought that I was doing right by the patients by giving them a Spanish-speaking physician. But now that you are telling me all of this, I am realizing that I wasn’t thinking about your wellbeing or learning.”
N (NEGOTIATE): Eduardo: “I understand, Mark. How do you feel about dividing the patients more equitably among the residents? It would also be an opportunity for my co-residents to work more with Spanish-speaking patients. And I’d like to see the hepatitis patient instead of the COPD patient, too, if that’s okay.”
Mark: “Absolutely, Eduardo. I’m sorry again.”
D (DETERMINE): Eduardo: “It is alright. We can be more communicative in the future to make sure we are on the same page, okay?”
Mark: “Definitely. I’ll ask for your input and listen to any concerns you have. Please don’t hesitate to speak up again if something is bothering you.

## Slide 24
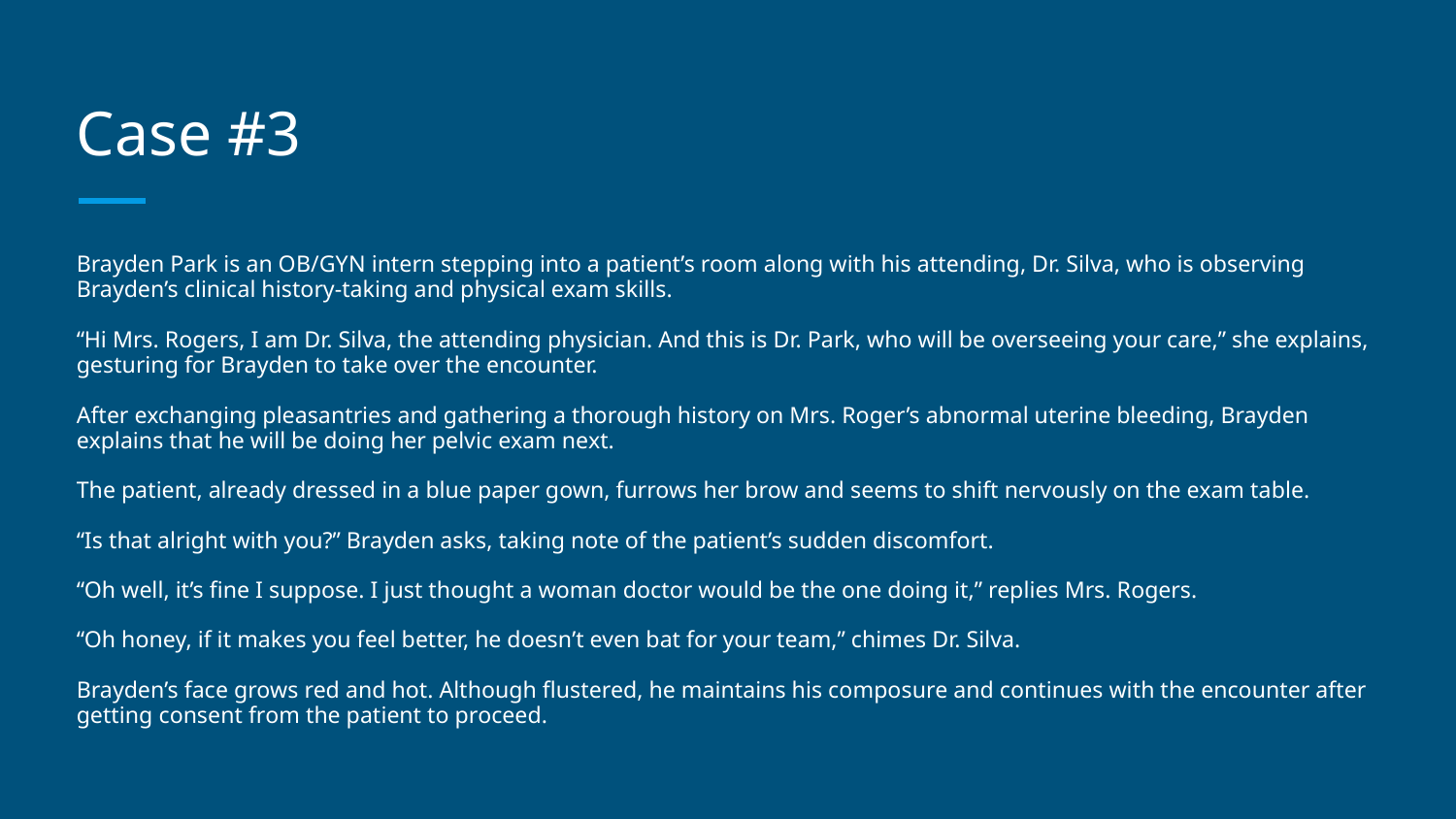

# Case #3
Brayden Park is an OB/GYN intern stepping into a patient’s room along with his attending, Dr. Silva, who is observing Brayden’s clinical history-taking and physical exam skills.
“Hi Mrs. Rogers, I am Dr. Silva, the attending physician. And this is Dr. Park, who will be overseeing your care,” she explains, gesturing for Brayden to take over the encounter.
After exchanging pleasantries and gathering a thorough history on Mrs. Roger’s abnormal uterine bleeding, Brayden explains that he will be doing her pelvic exam next.
The patient, already dressed in a blue paper gown, furrows her brow and seems to shift nervously on the exam table.
“Is that alright with you?” Brayden asks, taking note of the patient’s sudden discomfort.
“Oh well, it’s fine I suppose. I just thought a woman doctor would be the one doing it,” replies Mrs. Rogers.
“Oh honey, if it makes you feel better, he doesn’t even bat for your team,” chimes Dr. Silva.
Brayden’s face grows red and hot. Although flustered, he maintains his composure and continues with the encounter after getting consent from the patient to proceed.

## Slide 25
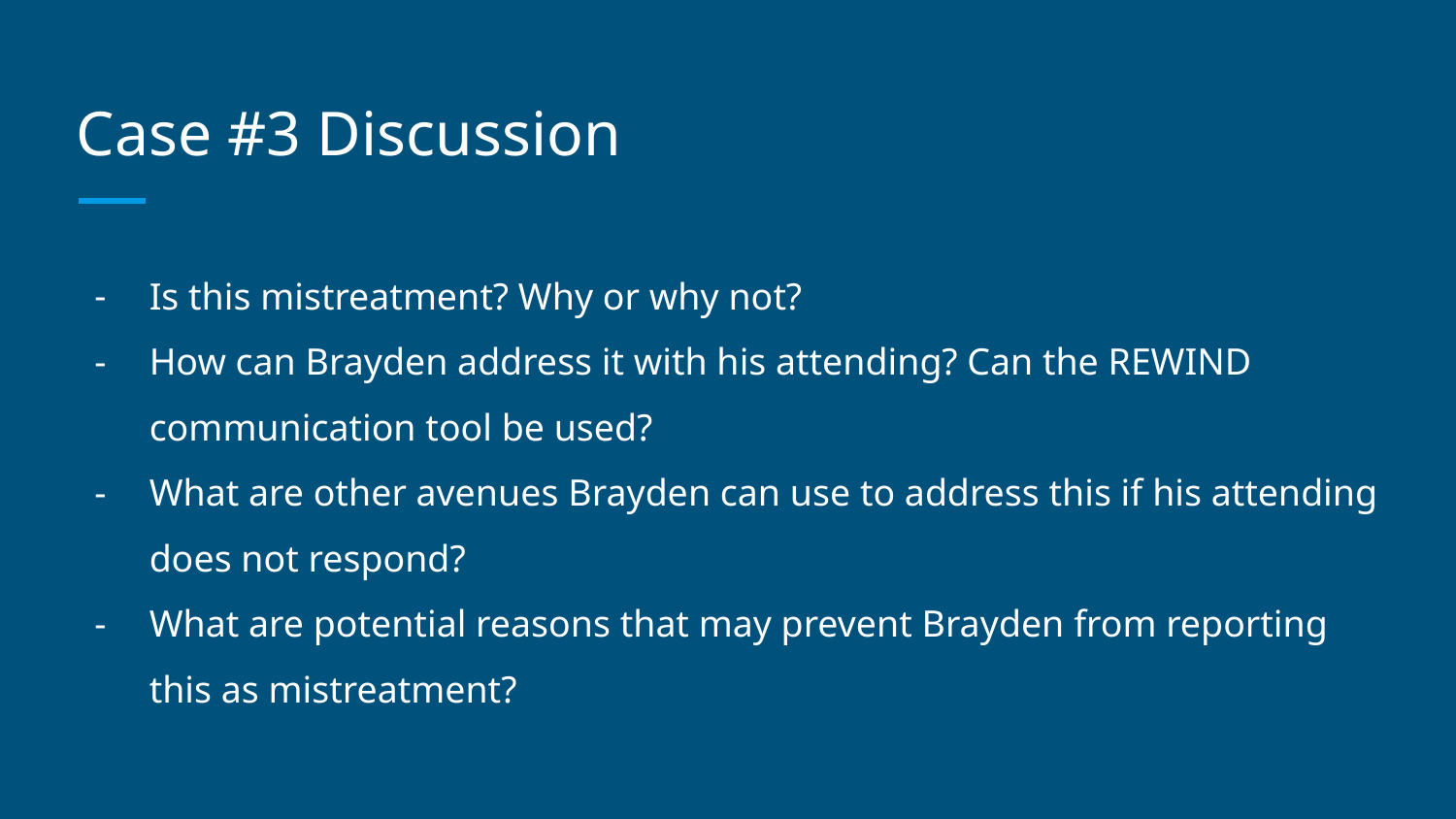

# Case #3 Discussion
Is this mistreatment? Why or why not?
How can Brayden address it with his attending? Can the REWIND communication tool be used?
What are other avenues Brayden can use to address this if his attending does not respond?
What are potential reasons that may prevent Brayden from reporting this as mistreatment?

## Slide 26
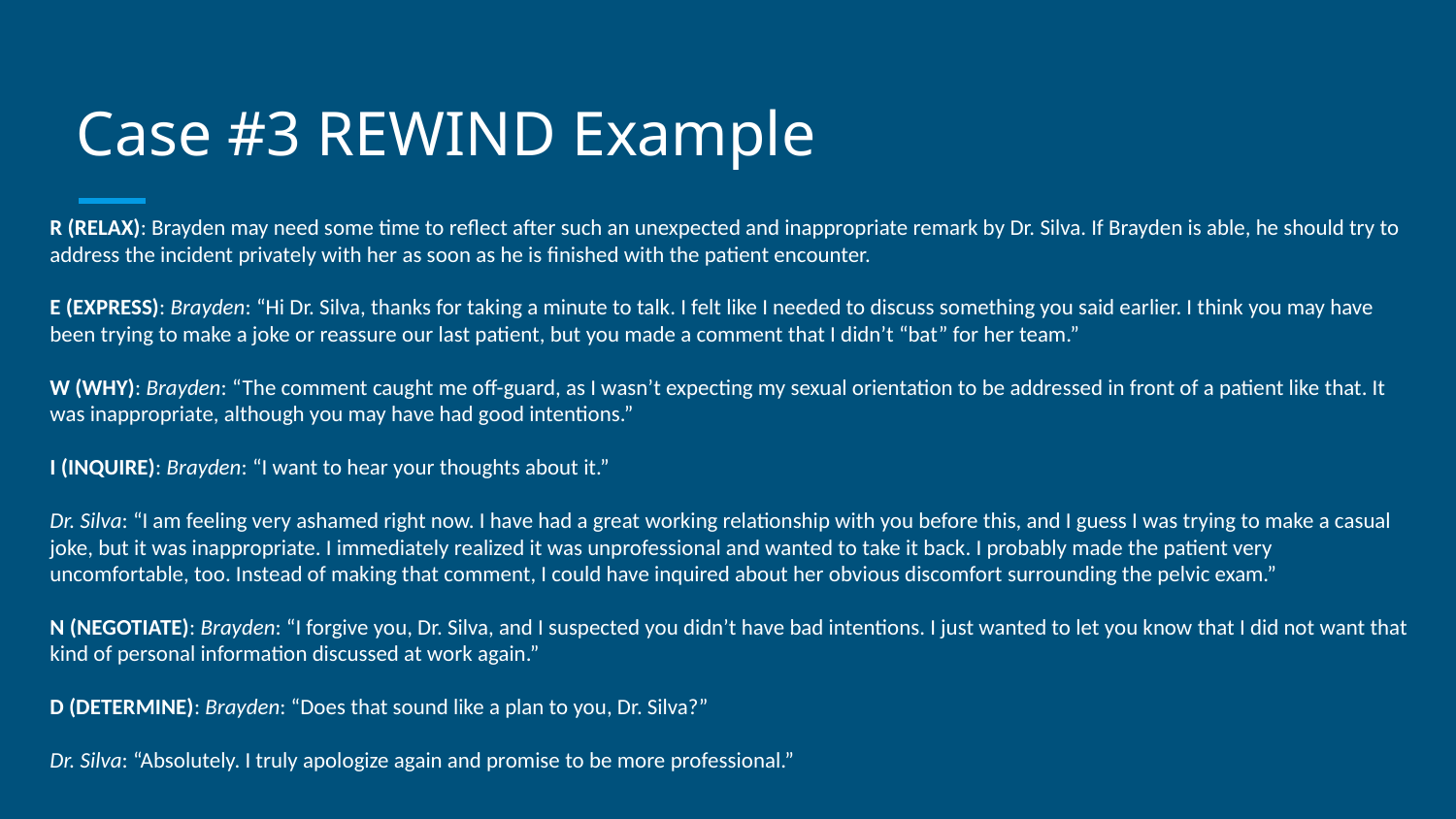

# Case #3 REWIND Example
R (RELAX): Brayden may need some time to reflect after such an unexpected and inappropriate remark by Dr. Silva. If Brayden is able, he should try to address the incident privately with her as soon as he is finished with the patient encounter.
E (EXPRESS): Brayden: “Hi Dr. Silva, thanks for taking a minute to talk. I felt like I needed to discuss something you said earlier. I think you may have been trying to make a joke or reassure our last patient, but you made a comment that I didn’t “bat” for her team.”
W (WHY): Brayden: “The comment caught me off-guard, as I wasn’t expecting my sexual orientation to be addressed in front of a patient like that. It was inappropriate, although you may have had good intentions.”
I (INQUIRE): Brayden: “I want to hear your thoughts about it.”
Dr. Silva: “I am feeling very ashamed right now. I have had a great working relationship with you before this, and I guess I was trying to make a casual joke, but it was inappropriate. I immediately realized it was unprofessional and wanted to take it back. I probably made the patient very uncomfortable, too. Instead of making that comment, I could have inquired about her obvious discomfort surrounding the pelvic exam.”
N (NEGOTIATE): Brayden: “I forgive you, Dr. Silva, and I suspected you didn’t have bad intentions. I just wanted to let you know that I did not want that kind of personal information discussed at work again.”
D (DETERMINE): Brayden: “Does that sound like a plan to you, Dr. Silva?”
Dr. Silva: “Absolutely. I truly apologize again and promise to be more professional.”

## Slide 27
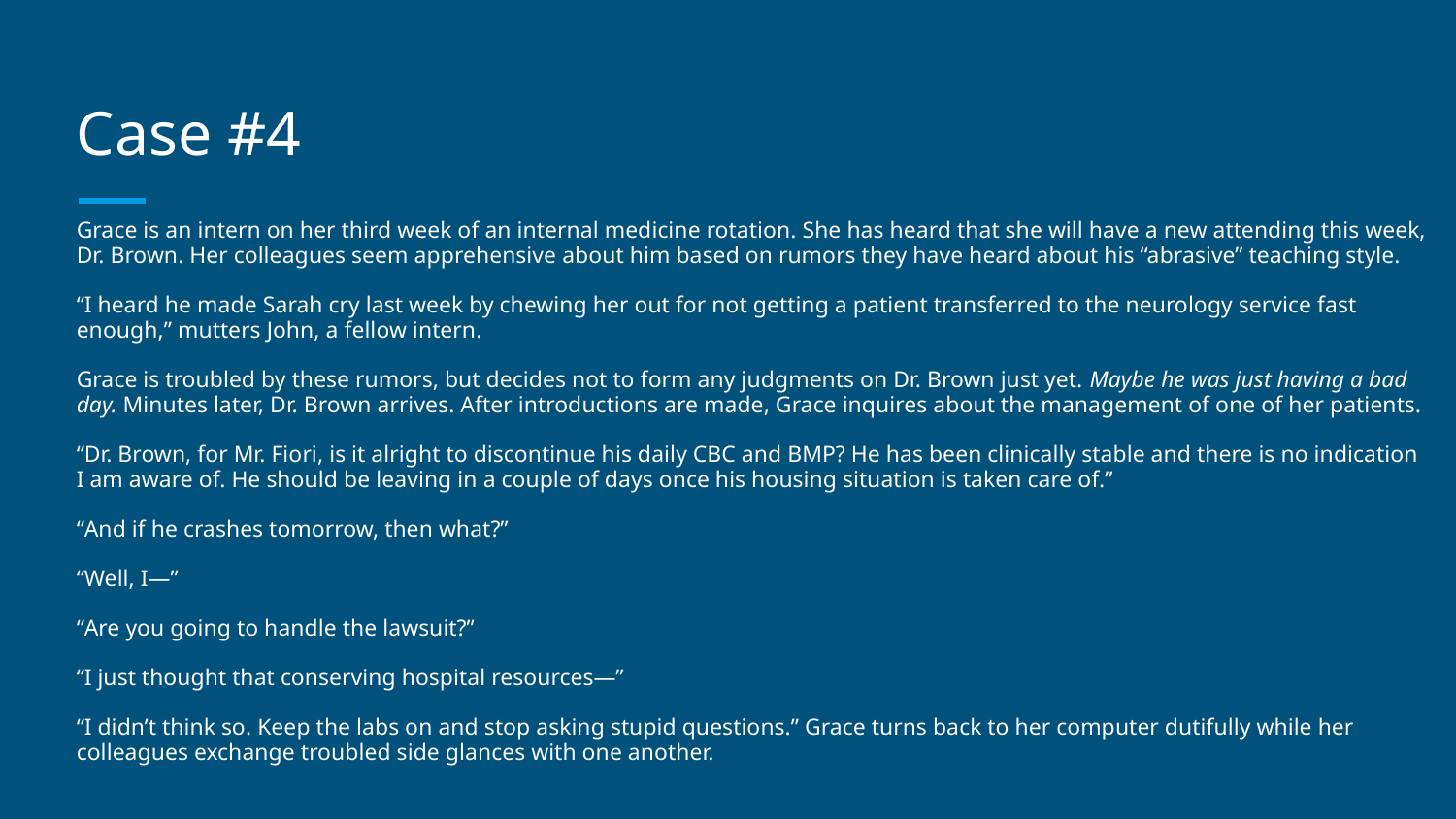

# Case #4
Grace is an intern on her third week of an internal medicine rotation. She has heard that she will have a new attending this week, Dr. Brown. Her colleagues seem apprehensive about him based on rumors they have heard about his “abrasive” teaching style.
“I heard he made Sarah cry last week by chewing her out for not getting a patient transferred to the neurology service fast enough,” mutters John, a fellow intern.
Grace is troubled by these rumors, but decides not to form any judgments on Dr. Brown just yet. Maybe he was just having a bad day. Minutes later, Dr. Brown arrives. After introductions are made, Grace inquires about the management of one of her patients.
“Dr. Brown, for Mr. Fiori, is it alright to discontinue his daily CBC and BMP? He has been clinically stable and there is no indication I am aware of. He should be leaving in a couple of days once his housing situation is taken care of.”
“And if he crashes tomorrow, then what?”
“Well, I—”
“Are you going to handle the lawsuit?”
“I just thought that conserving hospital resources—”
“I didn’t think so. Keep the labs on and stop asking stupid questions.” Grace turns back to her computer dutifully while her colleagues exchange troubled side glances with one another.

## Slide 28
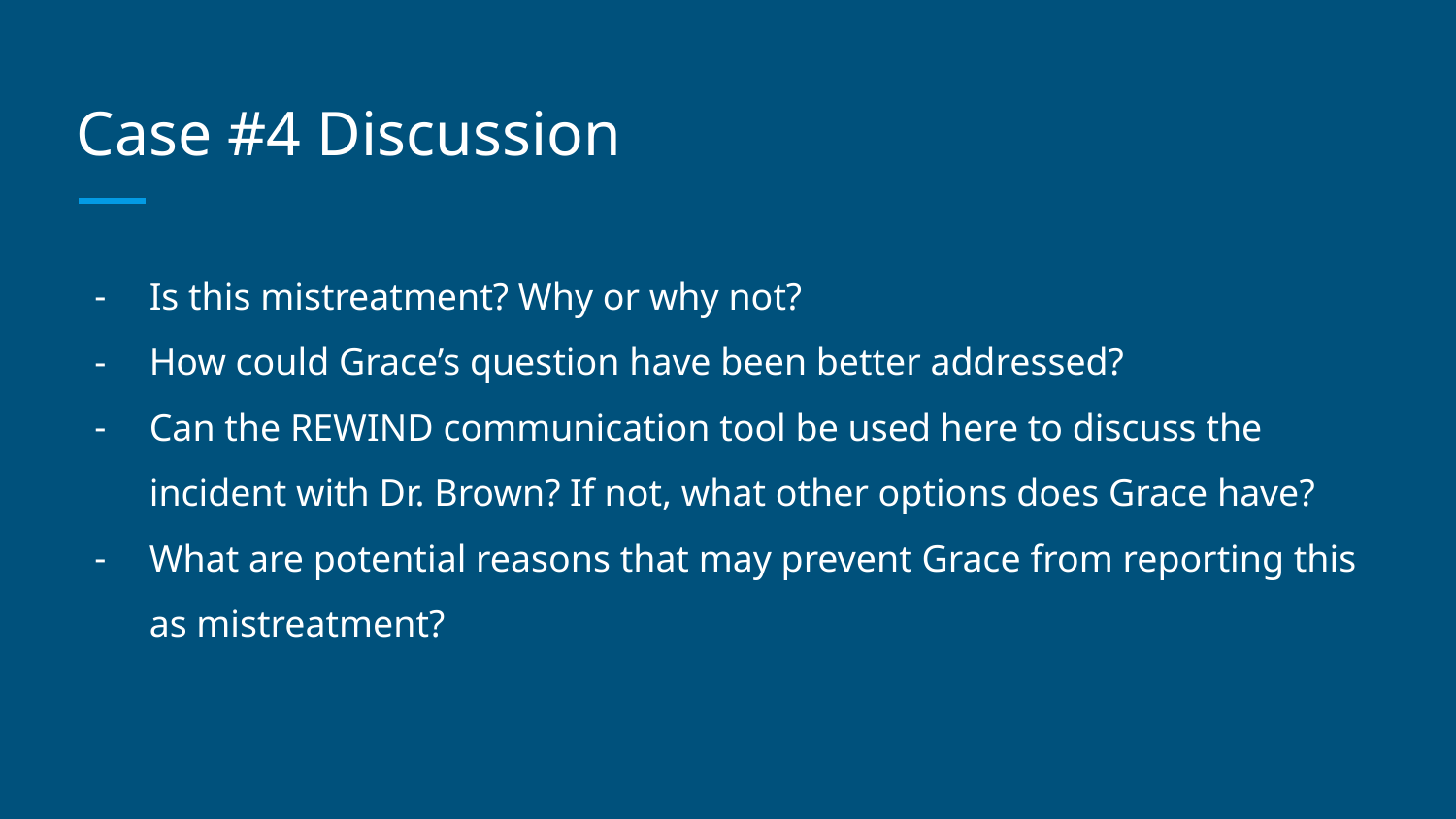

# Case #4 Discussion
Is this mistreatment? Why or why not?
How could Grace’s question have been better addressed?
Can the REWIND communication tool be used here to discuss the incident with Dr. Brown? If not, what other options does Grace have?
What are potential reasons that may prevent Grace from reporting this as mistreatment?

## Slide 29
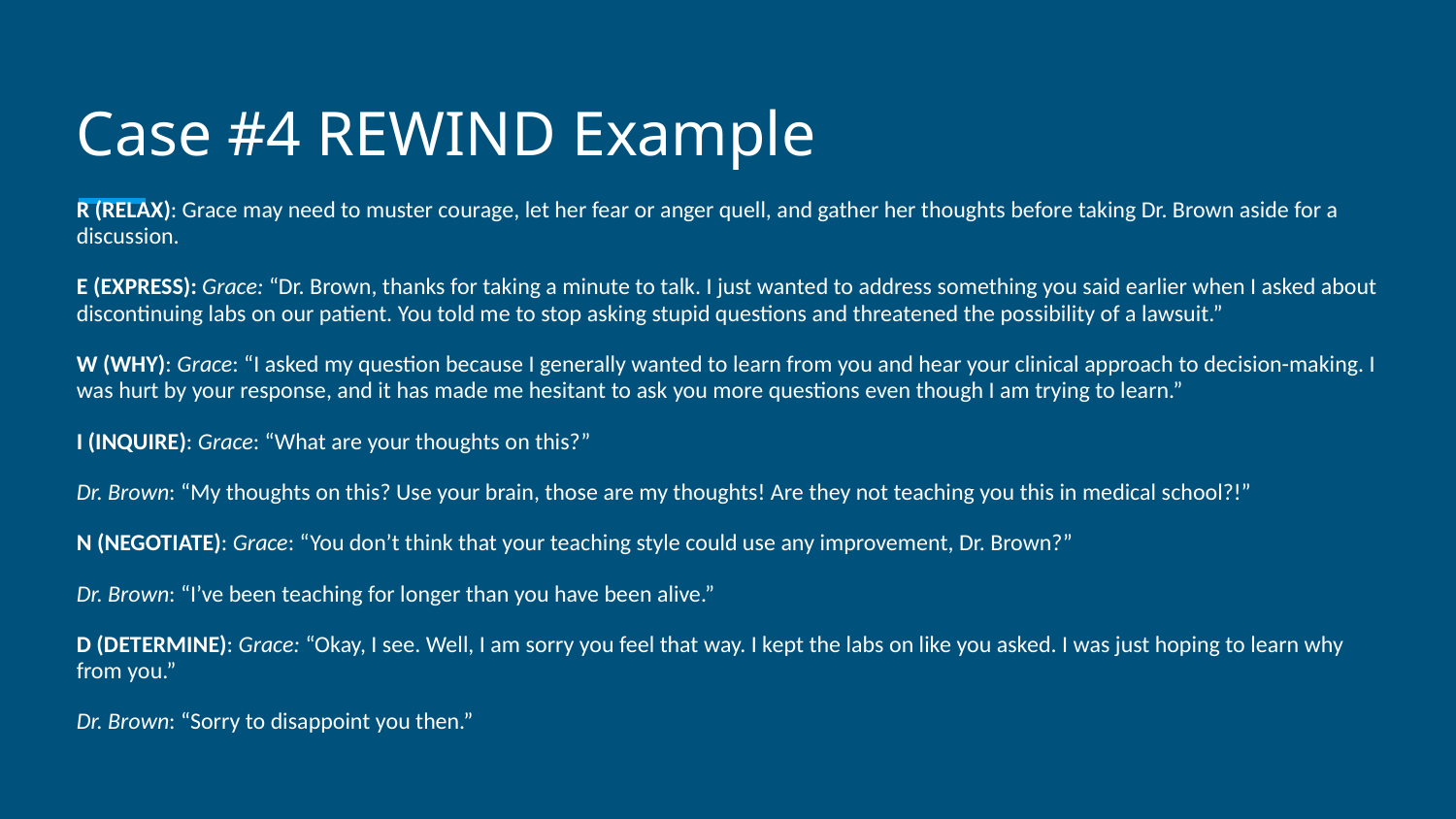

# Case #4 REWIND Example
R (RELAX): Grace may need to muster courage, let her fear or anger quell, and gather her thoughts before taking Dr. Brown aside for a discussion.
E (EXPRESS): Grace: “Dr. Brown, thanks for taking a minute to talk. I just wanted to address something you said earlier when I asked about discontinuing labs on our patient. You told me to stop asking stupid questions and threatened the possibility of a lawsuit.”
W (WHY): Grace: “I asked my question because I generally wanted to learn from you and hear your clinical approach to decision-making. I was hurt by your response, and it has made me hesitant to ask you more questions even though I am trying to learn.”
I (INQUIRE): Grace: “What are your thoughts on this?”
Dr. Brown: “My thoughts on this? Use your brain, those are my thoughts! Are they not teaching you this in medical school?!”
N (NEGOTIATE): Grace: “You don’t think that your teaching style could use any improvement, Dr. Brown?”
Dr. Brown: “I’ve been teaching for longer than you have been alive.”
D (DETERMINE): Grace: “Okay, I see. Well, I am sorry you feel that way. I kept the labs on like you asked. I was just hoping to learn why from you.”
Dr. Brown: “Sorry to disappoint you then.”

## Slide 30
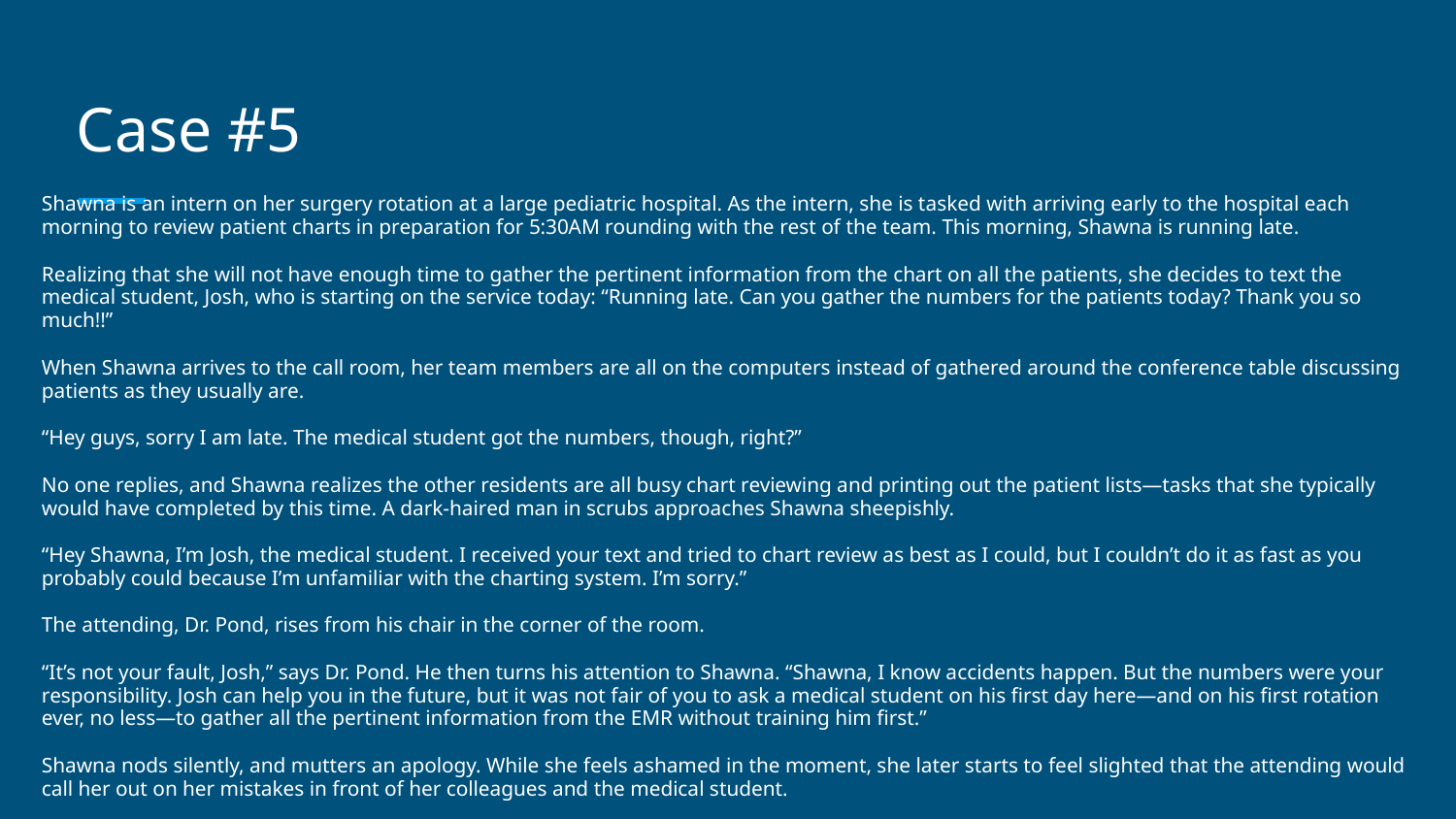

# Case #5
Shawna is an intern on her surgery rotation at a large pediatric hospital. As the intern, she is tasked with arriving early to the hospital each morning to review patient charts in preparation for 5:30AM rounding with the rest of the team. This morning, Shawna is running late.
Realizing that she will not have enough time to gather the pertinent information from the chart on all the patients, she decides to text the medical student, Josh, who is starting on the service today: “Running late. Can you gather the numbers for the patients today? Thank you so much!!”
When Shawna arrives to the call room, her team members are all on the computers instead of gathered around the conference table discussing patients as they usually are.
“Hey guys, sorry I am late. The medical student got the numbers, though, right?”
No one replies, and Shawna realizes the other residents are all busy chart reviewing and printing out the patient lists—tasks that she typically would have completed by this time. A dark-haired man in scrubs approaches Shawna sheepishly.
“Hey Shawna, I’m Josh, the medical student. I received your text and tried to chart review as best as I could, but I couldn’t do it as fast as you probably could because I’m unfamiliar with the charting system. I’m sorry.”
The attending, Dr. Pond, rises from his chair in the corner of the room.
“It’s not your fault, Josh,” says Dr. Pond. He then turns his attention to Shawna. “Shawna, I know accidents happen. But the numbers were your responsibility. Josh can help you in the future, but it was not fair of you to ask a medical student on his first day here—and on his first rotation ever, no less—to gather all the pertinent information from the EMR without training him first.”
Shawna nods silently, and mutters an apology. While she feels ashamed in the moment, she later starts to feel slighted that the attending would call her out on her mistakes in front of her colleagues and the medical student.

## Slide 31
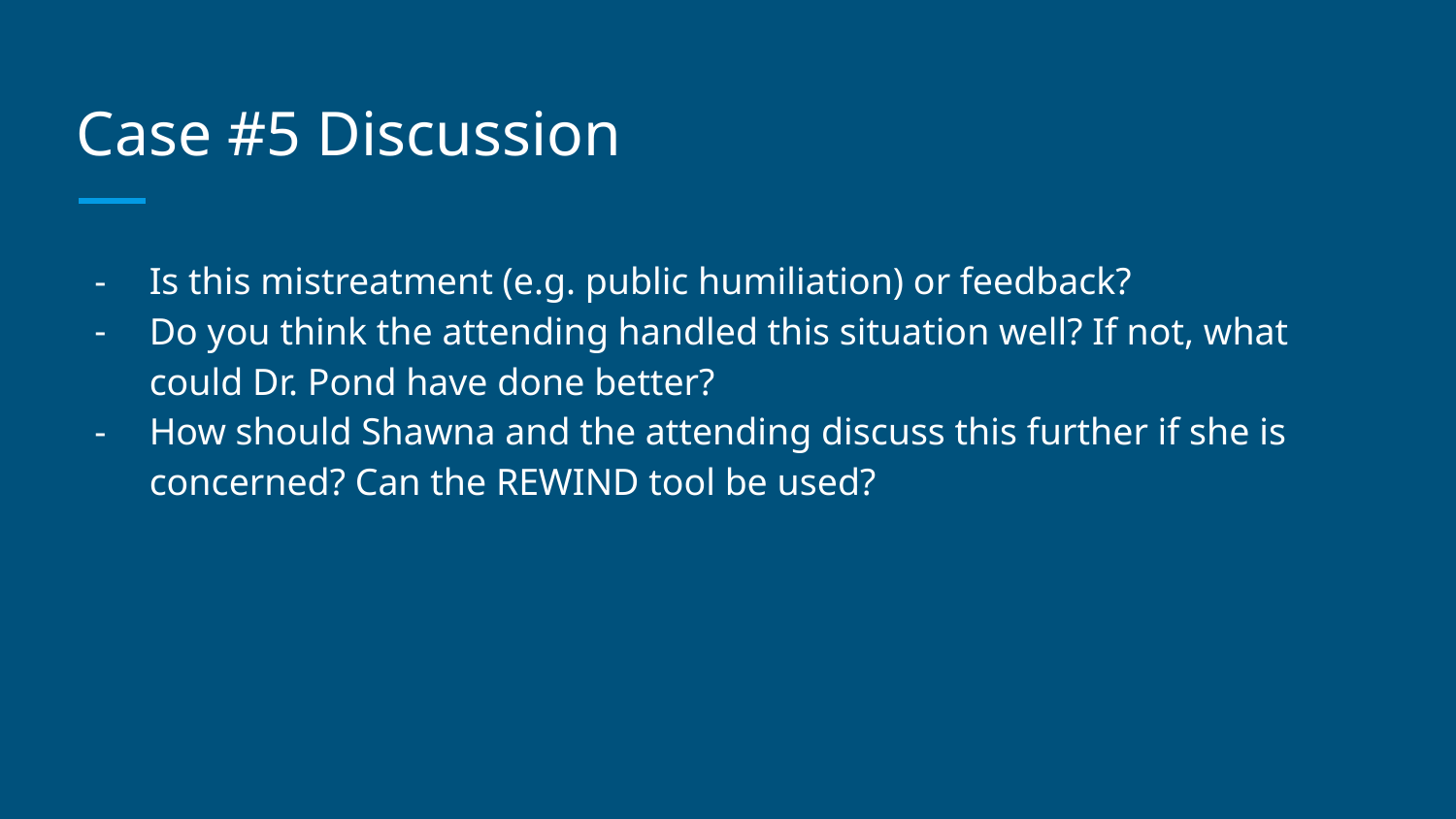

# Case #5 Discussion
Is this mistreatment (e.g. public humiliation) or feedback?
Do you think the attending handled this situation well? If not, what could Dr. Pond have done better?
How should Shawna and the attending discuss this further if she is concerned? Can the REWIND tool be used?

## Slide 32
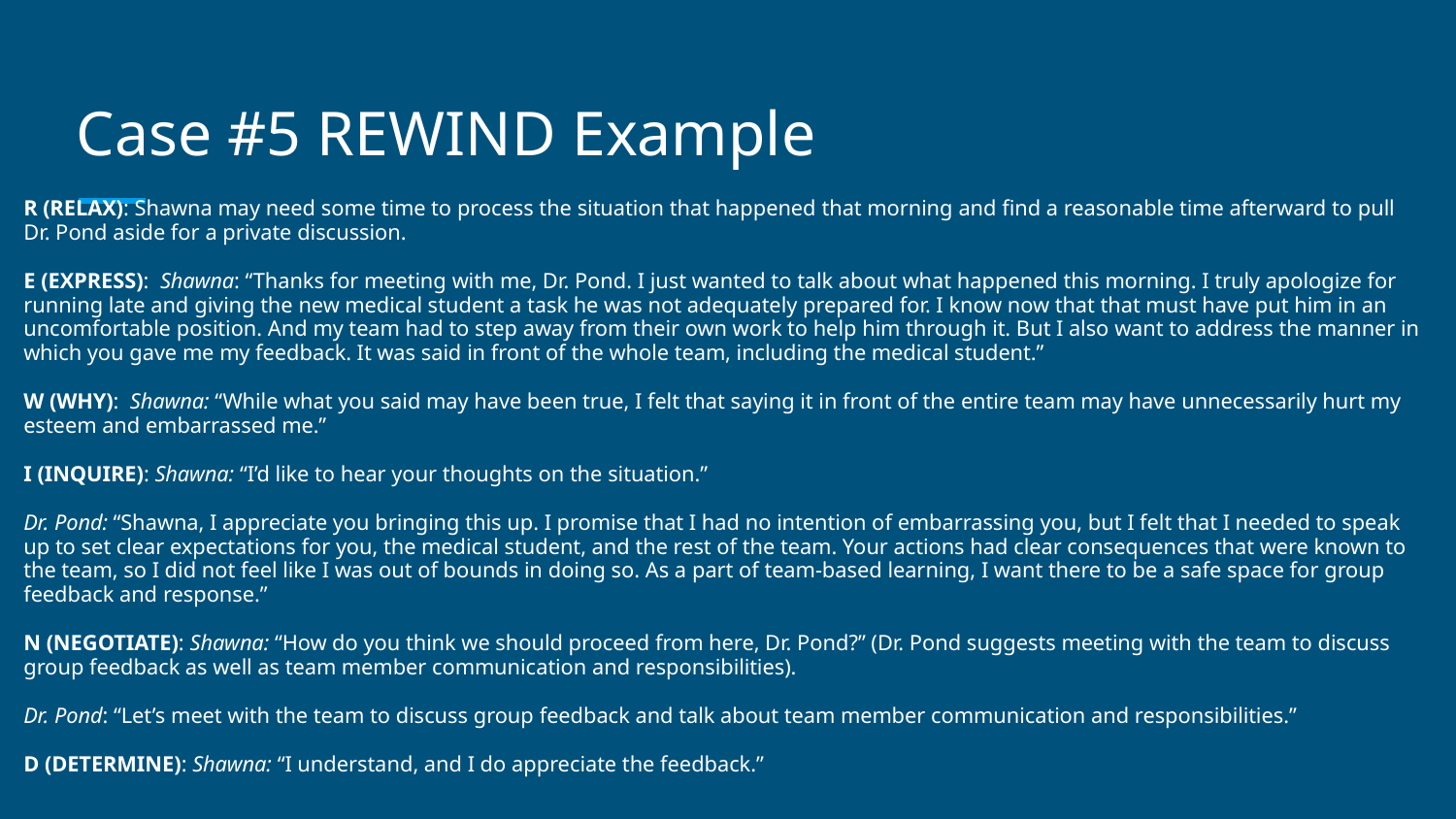

# Case #5 REWIND Example
R (RELAX): Shawna may need some time to process the situation that happened that morning and find a reasonable time afterward to pull Dr. Pond aside for a private discussion.
E (EXPRESS): Shawna: “Thanks for meeting with me, Dr. Pond. I just wanted to talk about what happened this morning. I truly apologize for running late and giving the new medical student a task he was not adequately prepared for. I know now that that must have put him in an uncomfortable position. And my team had to step away from their own work to help him through it. But I also want to address the manner in which you gave me my feedback. It was said in front of the whole team, including the medical student.”
W (WHY): Shawna: “While what you said may have been true, I felt that saying it in front of the entire team may have unnecessarily hurt my esteem and embarrassed me.”
I (INQUIRE): Shawna: “I’d like to hear your thoughts on the situation.”
Dr. Pond: “Shawna, I appreciate you bringing this up. I promise that I had no intention of embarrassing you, but I felt that I needed to speak up to set clear expectations for you, the medical student, and the rest of the team. Your actions had clear consequences that were known to the team, so I did not feel like I was out of bounds in doing so. As a part of team-based learning, I want there to be a safe space for group feedback and response.”
N (NEGOTIATE): Shawna: “How do you think we should proceed from here, Dr. Pond?” (Dr. Pond suggests meeting with the team to discuss group feedback as well as team member communication and responsibilities).
Dr. Pond: “Let’s meet with the team to discuss group feedback and talk about team member communication and responsibilities.”
D (DETERMINE): Shawna: “I understand, and I do appreciate the feedback.”

## Slide 33
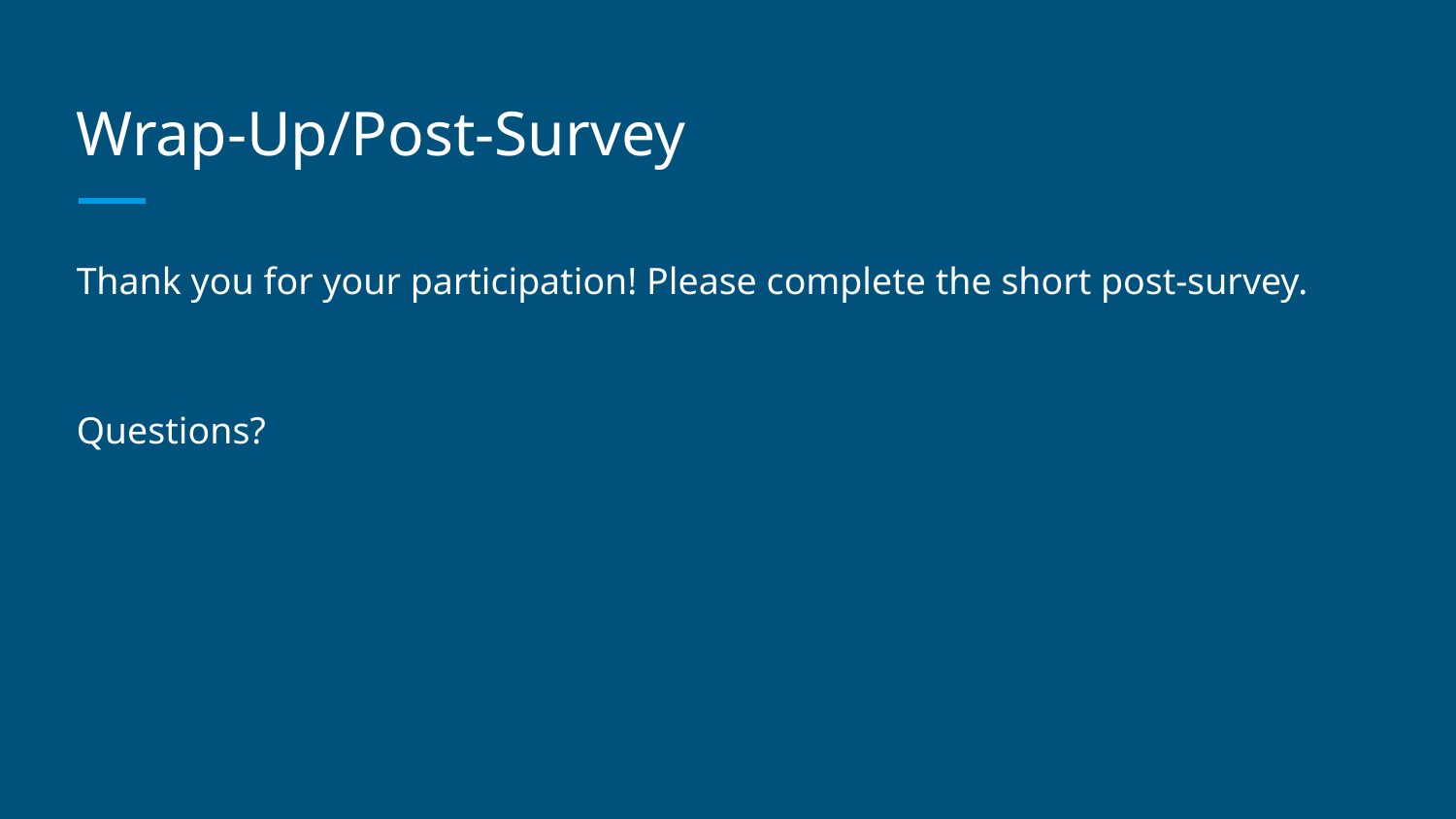

# Wrap-Up/Post-Survey
Thank you for your participation! Please complete the short post-survey.
Questions?
