# Supplementary material for: Mistreatment in Residency: Intervening With the REWIND Communication Tool
Source: MedEdPORTAL. 2022 Apr 26;18:11245. doi: 10.15766/mep_2374-8265.11245 (PMC9038987; doi:10.15766/mep_2374-8265.11245)
Supplement: Supplementary file 1 — Mistreatment in Residency.pptxWorkshop Presurvey.docxWorkshop Postsurvey.docxFacilitator Guide.docxREWIND Handout.docxCase 2 Handout.docxCase 3 Handout.docxCase 4 Handout.docxCase 5 Handout.docx [file mep_2374-8265.11245-s001.zip › B. Workshop Presurvey.docx]

Mistreatment in Residency: Intervening with the REWIND Communication Tool: **Pre-Workshop Evaluation**

**Before** completing today’s workshop, how would you rate your ability to:

|  | **Not at all proficient** |  |  |  | **Extremely Proficient** |
| --- | --- | --- | --- | --- | --- |
| 1. Define mistreatment in medical education | 1 | 2 | 3 | 4 | 5 |
| 1. Identify the resources for reporting mistreatment at your institution | 1 | 2 | 3 | 4 | 5 |
| 1. Report mistreatment in medical education | 1 | 2 | 3 | 4 | 5 |
| 1. Respond directly to mistreatment that is **directed towards you** | 1 | 2 | 3 | 4 | 5 |
| 1. Respond directly to mistreatment that **you have observed** | 1 | 2 | 3 | 4 | 5 |
| 1. Utilize the REWIND communication tool to respond to mistreatment | 1 | 2 | 3 | 4 | 5 |

What is your level of clinical training?

- Pre-Med
- Medical Student
- Resident
- Faculty
- Other ______________________________

What is your gender/gender identity? Select all that apply.

- Man
- Woman
- Transgender
- Non-binary
- Prefer to self-describe ______________________________
- Prefer not to say

Do you consider yourself to be of Latino, Hispanic, and/or of Spanish origin?

- Yes
- No
- Prefer not to say

How do you describe your race? Select all that apply.

- American Indian or Alaska Native
- Asian
- Black or African American
- Middle Eastern or North African
- Native Hawaiian or other Pacific Islander
- White
- Prefer to self-describe____________________
- Prefer not to say

How do you identify your sexual orientation? Select all that apply.

- Straight/heterosexual
- Lesbian
- Gay
- Bisexual
- Asexual
- Prefer to self-describe____________________
- Prefer not to say

**Mistreatment** is defined as **behavior that shows disrespect for the dignity of others** and **unreasonably interferes with the learning process**.

| The following questions ask about mistreatment that **you have personally experienced** during **your medical education**. |
| --- |

Have you **experienced mistreatment** within medical education?

- Yes
- No
- Not sure

What **types of mistreatment** have you previously **experienced** within medical education? (Select all that apply)

- Verbal abuse
- Public humiliation
- Threatened with and/or subjected to physical harm
- Neglected and/or left out
- Made to perform physical services
- Threatened with retaliation or retaliated against
- Subjected to unwanted sexual advances
- Mistreated based on gender or gender identity
- Mistreated based on race and/or ethnicity
- Mistreated based on sexual orientation
- Mistreated based on disability
- Subjected to offensive behaviors based on personal beliefs or personal characteristics other than gender, race/ethnicity, sexual orientation, or disability
- Other ________________________________________

Who has been the **source of mistreatment** that you have **experienced** within medical education? (Select all that apply)

- Patients
- Staff
- Students
- Residents
- Faculty
- Other ______________________________

| The following questions ask about mistreatment you have **witnessed or observed within medical education.** |
| --- |

Have you **witnessed mistreatment** within medical education?

- Yes
- No
- Not sure

What **types of mistreatment** have you previously **witnessed** within medical education? (Select all that apply)

- Verbal abuse
- Public humiliation
- Threatened with and/or subjected to physical harm
- Neglected and/or left out
- Made to perform physical services
- Threatened with retaliation or retaliated against
- Subjected to unwanted sexual advances
- Mistreated based on gender or gender identity
- Mistreated based on race and/or ethnicity
- Mistreated based on sexual orientation
- Mistreated based on disability
- Subjected to offensive behaviors based on personal beliefs or personal characteristics other than gender, race/ethnicity, sexual orientation, or disability
- Other ________________________________________

Who has been the **source of mistreatment** that you have **witnessed** within medical education? (Select all that apply)

- Patients
- Staff
- Students
- Residents
- Faculty
- Other ______________________________
